# Evaluation of PD‐L1 Expression in Patients With Non–Small Cell Lung Cancer Using DCE‐MRI Quantitative Analysis

**DOI:** 10.1111/crj.70125

**Published:** 2025-09-27

**Authors:** Chen Yang, Fandong Zhu, Qianling Li, Chenwen Sun, Hongyan Jin, Zhenhua Zhao

**Affiliations:** ^1^ Department of Radiology Shaoxing People's Hospital Shaoxing China; ^2^ Department of Pathology Shaoxing People's Hospital Shaoxing China

**Keywords:** histogram, magnetic resonance imaging, non–small cell lung cancer, programmed death ligand‐1, quantitative analysis

## Abstract

**Purpose:**

The aim is to evaluate the expression of programmed death ligand 1 (PD‐L1) in patients with non–small cell lung cancer (NSCLC) using quantitative perfusion parameters based on dynamic contrast‐enhanced magnetic resonance imaging (DCE‐MRI).

**Methods:**

A total of 35 patients with a confirmed diagnosis of NSCLC and sufficient tissue pathology were enrolled in the study. The immunohistochemical (IHC) results were used as the gold standard to determine the thresholds for grouping the patients. The patients were divided into three categories based on their PD‐L1 expression: (1) PD‐L1‐negative (IHC < 1%) and PD‐L1‐positive (IHC ≥ 1%); (2) PD‐L1 weak (IHC < 50%) and strong expression (IHC ≥ 50%); and (3) PD‐L1 nonexpression (IHC < 1%), low expression (IHC between 1% and 49%), and high expression (IHC ≥ 50%). DCE‐MRI datasets were analyzed to acquire histogram parameters, including mean value, uniformity, skewness, kurtosis, entropy, energy, and quantity, of quantitative perfusion parameters using the extended Tofts model (ETM) and the exchange model (ECM). Subsequently, the parameters were compared between the aforementioned groups.

**Results:**

IHC showed PD‐L1 < 1% in 20 cases, PD‐L1 (1%–49%) in 14 cases, and PD‐L1 ≥ 50% in 14 cases. At a threshold of 50%, statistically significant differences were observed for ETM/*K* ^trans^ (*Q*25 and *Q*50), ETM/*K*_ep_ (*Q*10), and ECM/*V*_e_ (*Q*75 and *Q*90), with values being higher in the weak PD‐L1 expression group. With thresholds of 1% and 50%, the results of the pairwise comparison showed that the ECM/*V*_e_ (*Q*75) value in the low PD‐L1 expression group was significantly higher than that in the high PD‐L1 expression group.

**Conclusion:**

DCE‐MRI quantitative analysis is a valuable tool for the evaluation of PD‐L1 expression in NSCLC. It provides a noninvasive method that can be employed as an adjunctive technique for the stratification of PD‐L1 expression in patients with NSCLC.

## Graphical abstract

The expression of PD‐L1 in NSCLC may be related to the hemodynamics of the lesion. The study utilized quantitative perfusion parameters such as *K* ^trans^, *K*_ep_, *V*_e_, *V*_p_, and *F*_p_ with their histogram parameters (mean value, uniformity, skewness, kurtosis, entropy, energy, and quantity) from the extended Tofts model (ETM) and the exchange model (ECM) to assess the expression of PD‐L1. The values of ETM/*K* ^trans^ (*Q*25 and *Q*50), ETM/*K*_ep_ (*Q*10), and ECM/*V*_e_ (*Q*75 and *Q*90) were higher in the weak PD‐L1 expression group (< 50%) compared to the strong PD‐L1 expression group (≥ 50%). Besides, ECM/*V*_e_ (*Q*75) was statistically different between the three classification PD‐L1 expression groups with thresholds of 1% and 50%. These results thus presented that DCE‐MRI quantitative analysis provides a noninvasive method for the evaluation of PD‐L1 in NSCLC.

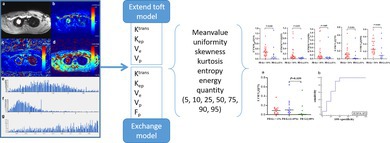

## 1 Introduction

The reported 5‐year overall survival (OS) of non–small cell lung cancer (NSCLC) is approximately 25% [1]. In recent years, the advent of immunotherapy has had a transformative effect on the treatment outlook for patients with NSCLC [2]. Immunotherapy targets pivotal mechanisms within the tumor microenvironment, such as programmed death ligand‐1 (PD‐L1). This is highly expressed on tumor cells and binds to PD‐1, thereby promoting the apoptosis of cytotoxic T lymphocytes [3]. It is of the utmost importance to conduct regular assessments of PD‐L1 expression. In clinical trials, some patients showed initial sensitivity to anti‐PD‐L1 antibodies, followed by the emergence of resistance and ultimately ineffective treatment, which resulted in tumor progression. Four Food and Drug Administration (FDA)‐approved immunohistochemistry (IHC) methods have been used to assess PD‐L1 expression [4]. However, it should be noted that the samples used for IHC must be obtained by surgery or puncture and are therefore unsuitable for dynamic monitoring during treatment. Furthermore, due to the internal heterogeneity of tumors, some samples used may not fully reflect a specific tumor or represent multiple tumors [5]. It is therefore necessary to develop a noninvasive diagnostic method that can comprehensively and dynamically evaluate PD‐L1 expression in order to facilitate the screening of patients suitable for immunotherapy and to dynamically monitor the effect of immunotherapy.

PD‐L1 is intimately linked with tumor hemodynamics. It has been proposed that anti‐angiogenic therapy may result in the upregulation of PD‐L1 expression [6, 7]. The inhibition of angiogenesis results in the development of ischemia and hypoxia within the tumor tissue, which in turn induces the expression of angiogenic factors and promotes pathological angiogenesis in tumors. Abnormal angiogenesis stimulates the upregulation of PD‐L1 expression on abnormal phenotype vascular endothelial cells and tumor cells [8].

The development of imaging techniques, particularly the application of quantitative perfusion technology, provides a feasible detection technology for the evaluation of drug targets in lung cancer [9, 10]. Dynamic contrast‐enhanced MRI (DCE‐MRI), which is free of radiation hazards and allows the acquisition of imaging data from multiple sequences, has demonstrated value in the assessment of lung disease [11, 12, 13, 14, 15, 16]. In evaluating the expression of proteins linked to tumor cell proliferation and immunoregulatory pathways, DCE‐MRI quantitative analysis of perfusion parameters and apparent diffusion coefficient (ADC) has been demonstrated to be a valuable approach [10, 17].

This study used DCE‐MRI quantitative analysis to evaluate PD‐L1 expression in lung cancer patients and extracted quantitative perfusion parameters associated with PD‐L1 expression in lung cancer tumors. This approach yielded providing noninvasive and repeatable imaging tools for dynamic monitoring of PD‐L1 expression in lung cancer.

## 2 Materials and Methods

### 2.1 Patients

Ethical approval was obtained for this study (Approval No. 202063). Informed consent for MRI examination was signed by each patient.

The study population comprised patients with suspected lung cancer (*n* = 124) who underwent chest MRI at our hospital between January 2018 and October 2022. The exclusion criteria for the study were as follows: (1) Patients with pathological findings of lung tumors other than NSCLC (*n* = 23). (2) Patients who had received any treatment or invasive surgery prior to MRI (*n* = 8). (3) Patients with insufficient remaining pathological tissue for PD‐L1 immunohistochemical detection (*n* = 42). (4) Patients whose scans were of poor quality or could not clearly show tumor boundaries (*n* = 5).

The study population was ultimately comprised of 35 patients, including 21 with adenocarcinomas (ACs) and 14 with squamous cell carcinomas (SCCs).

### 2.2 MRI Scanning

The patients were able to breathe freely. The images were acquired using a 3.0 T MR scanner (Siemens Bio, Germany). The scan sequences included T2‐weighted imaging (T2WI), T1‐weighted imaging (T1WI) with volume‐interpolated breath‐hold examination (VIBE) fat suppression, diffusion‐weighted imaging, and ADC sequences. The setup parameters were as follows: repetition time/echo time = 3.25/1.17 ms; field of view = 350 × 282 mm^2^; matrix size = 288 × 162; slice width = 5 mm; number of slices = 30; flip angles = 5°, 10°, and 15°; and *b* value = 800 s/mm^2^. The dynamic enhancement scans, based on 3D VIBE T1WI, employed 35 time phases, with a total time phase of 227.5 s. The contrast agent utilized was gadolinium diamine, at a concentration of 0.1 mmol/kg, with an injection rate of 2–3 mL/s. The parameters of the dynamic enhancement scans were as follows: flip angle = 10°, temporal resolution = 6.5 s, and voxel = 1.7 mm × 1.2 mm × 5.0 mm. The remaining parameters were identical to those used for the plain scans.

### 2.3 Imaging Data Processing

**FIGURE 1:**
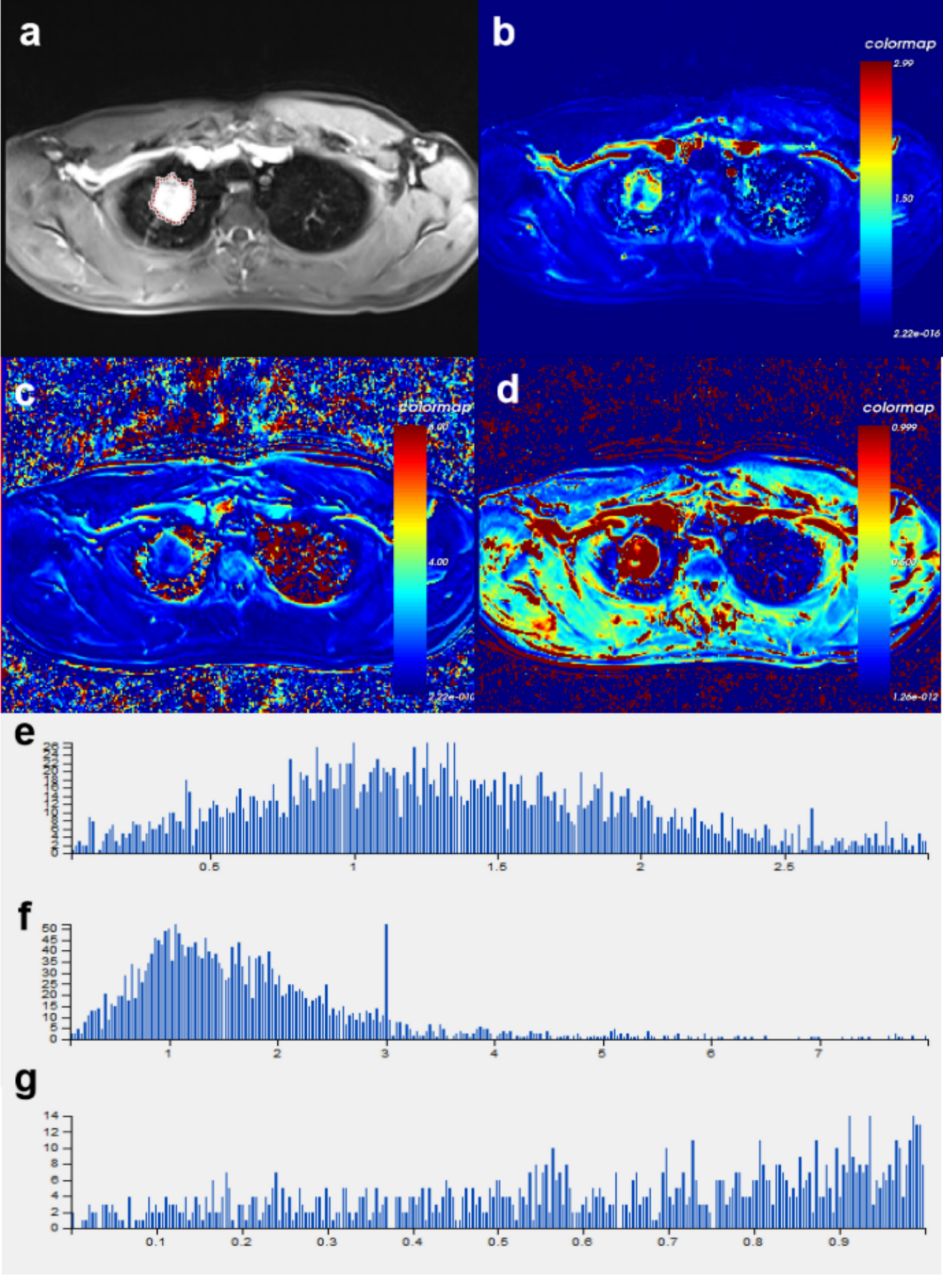
(a) An AC patient with high PD‐L1 expression. (b–d) Pseudocolor plots of *K* ^trans^, *K*_ep_, and *V*_e_ in that order (all from ETM). (e) The histogram parameters of *K* ^trans^ are as follows: mean value = 0.085, skewness = 2.095, kurtosis = 13.475, uniformity = 0.260, energy = 0.016, entropy = 6.117, *Q*5 = 0.010, *Q*10 = 0.017, *Q*25 = 0.036, *Q*50 = 0.075, *Q*75 = 0.121, *Q*90 = 0.163, and *Q*95 = 0.187. (f) The histogram parameters of *K*_ep_ are as follows: mean value = 1.231, skewness = 2.598, kurtosis = 10.449, uniformity = 0.107, energy = 0.015, entropy = 5.435, *Q*5 = 0.053, *Q*10 = 0.247, *Q*25 = 0.570, *Q*50 = 0.958, *Q*75 = 1.549, *Q*90 = 2.414, and *Q*95 = 3.057. (g) The histogram parameters of *V*_e_ are as follows: mean value = 0.129, skewness = 3.856, kurtosis = 14.212, uniformity = −0.515, energy = 0.025, entropy = 5.554, *Q*5 = 0.014, *Q*10 = 0.023, *Q*25 = 0.046, *Q*50 = 0.088, *Q*75 = 0.118, *Q*90 = 0.141, and *Q*95 = 0.167.

Region of interest (ROI) mapping was performed by two residents who had received training in the use of the software and were supervised by a senior physician (Figure 1). To assess intraobserver reproducibility, Physician 1 measured the parameters twice, with an interval of 1 month between measurements. To assess intraobserver reproducibility, Physician 2 measured the parameters once. The final assessment was based on the mean of the three measurements.

The DCE‐MRI data were processed using Omni.haemodynamics software (GE, China). The motion artefacts of the images were calibrated using a three‐dimensional nonrigid alignment technique, which converts the time–signal curve into a time–concentration curve. The pre‐enhancement plasma T1 value was set to 1600 ms, the muscle T1 value to 1580 ms, and the erythrocyte‐specific volume was set to 0.42. In the Omni.haemodynamics software, the dual‐supply hemodynamic model (user AIF) was selected, and ROIs with a fixed diameter of 8 mm were drawn at the opening of the bronchial artery of the thoracic aorta and the main trunk of the affected pulmonary artery, respectively. Subsequently, the ROIs for the lesions were delineated by selecting three to five layers of nontumor necrotic and cystic areas while avoiding the inclusion of normal tissues. The hemodynamic parameters were calculated (using the exchange model [ECM] to obtain *K* ^trans^, *K*_ep_, *V*_e_, *V*_p_, and *F*_p_ and the extended Tofts model [ETM] to obtain *K* ^trans^, *K*_ep_, *V*_e_, and *V*_p_). The parameter definitions and units are detailed in Table 1. A total of 13 histogram parameters were extracted from each hemodynamic parameter, including mean value, uniformity, skewness, kurtosis, entropy, energy, and quantile (*Q*5, *Q*10, *Q*25, *Q*50, *Q*75, *Q*90, and *Q*95).

**TABLE 1:** Meaning and units of hemodynamic parameters.

Notation	Meaning	Unit
*K* ^trans^	Contrast agent flow transfer constants from intravascular to extravascular extracellular space (EES)	min^−1^
*K*_ep_	Rate constant for intravascular return of contrast from the EES	min^−1^
*V*_e_	EES volume fraction	% or mL/mL
*V*_p_	Plasma volume fraction	% or mL/mL
*F*_p_	Plasma flow rate through a unit vessel	mL/min/mL

### 2.4 PD‐L1 Analysis Method

A working solution of PD‐L1 concentrate (E1L3N) was used to detect PD‐L1 expression in pathological tissues. The pathological sections were reviewed independently by two physicians, and disagreements were discussed and resolved. PD‐L1 immunohistochemical staining was scored according to published methods [18]. The tumor proportion score (TPS) was calculated using the following formula:

$$\mathrm{TPS}=\left(\mathrm{PD}-L1-\text{positive tumor cells}/\text{the total live tumor cells}\right)\times 100\%.$$

**FIGURE 2:**
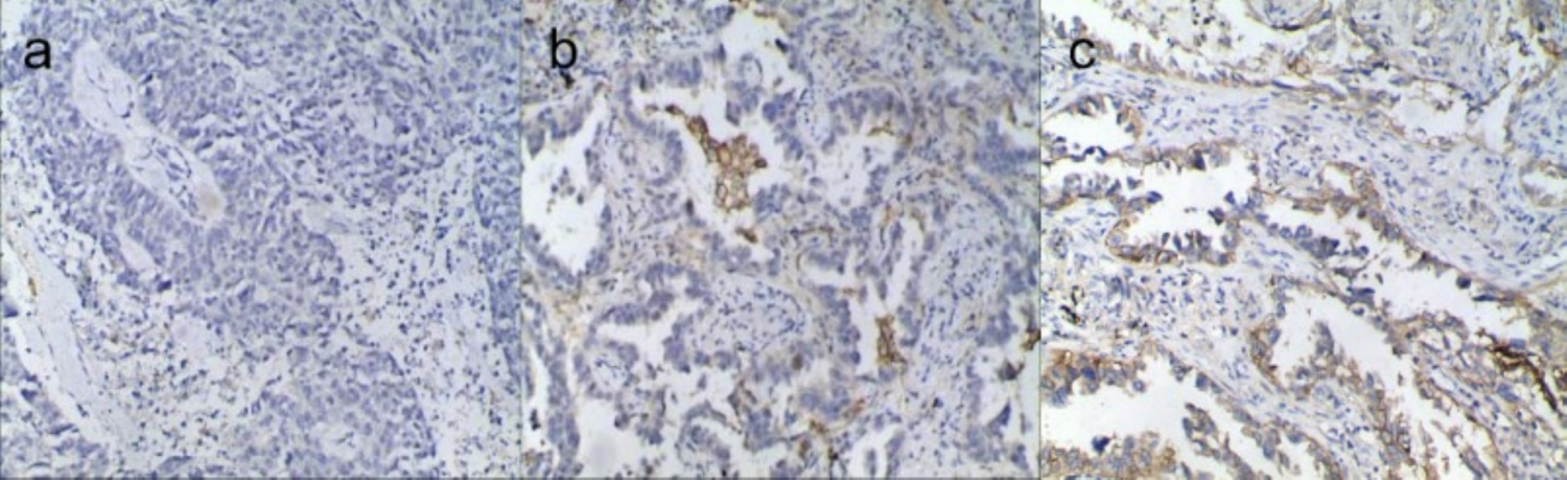
Immunohistochemical staining of PD‐L1 (×400). (a) A SCC patient with TPS < 1% (PD‐L1 nonexpression). (b) An AC patient with TPS 1%–49% (low PD‐L1 expression). (c) An AC patient with TPS ≥ 50% (high PD‐L1 expression).

The complete or partial circumferential cell membrane staining was defined as positive tumor cells. From the scoring process, cytoplasmic staining and tumor‐associated immune cells (e.g., macrophages) were excluded. PD‐L1‐positive expression (expression on tumor cells ≥ 50% or ≥ 1%) is one of the indications for ICIs (first or second line) alone in patients with advanced NSCLC according to the National Comprehensive Cancer Network (NCCN) and National Medical Products Administration (NMPA) guidelines [19, 20]. Patients exhibiting disparate levels of PD‐L1 expression achieve different gains in efficacy, with the PD‐L1 ≥ 50% group achieving a markedly superior degree of efficacy. Accordingly, three distinct PD‐L1 grouping thresholds were employed in this study to identify patients who would benefit from different treatment regimens and achieve varying degrees of efficacy gains. On the basis of the IHC results, a threshold of 1% was used to divide the tumors into two groups: PD‐L1‐negative (TPS < 1%) and PD‐L1‐positive (TPS ≥ 1%), a threshold of 50% was used to classify tumors into two groups: weak PD‐L1 expression (TPS < 50%) and strong PD‐L1 expression (TPS ≥ 50%). A threshold of 1% and 50% was used for classification of tumors into three groups (Figure 2): PD‐L1 nonexpression (TPS < 1%), low expression (TPS = 1%–49%), and high expression (TPS ≥ 50%).

### 2.5 Statistical Analyses

The data were analyzed using the SPSS 25.0 software (Version 25.0, Armonk, NY). The differences in DCE‐MRI quantitative parameters between the dichotomous data sets (PD‐L1‐negative and PD‐L1‐positive groups, weak and strong PD‐L1 expression groups) were compared using the Student's *t*‐test for normally distributed data and the Mann–Whitney *U*‐test otherwise. To compare the differences in DCE‐MRI quantitative parameters and ADC means among the three groups of PD‐L1 nonexpression, low expression, and high expression, the LSD‐*t* test was employed for data that satisfied both the normality and chi‐squared criteria. The Kruskal–Wallis (K‐W) rank sum test was used. The significance level was set at *p* < 0.05.

## 3 Results

### 3.1 Clinical Characteristics of Patients

A total of 35 patients with an age range of 50–85 years were included in this study. As shown in Table 2, the various thresholds of PD‐L1 expression were not statistically different between AC and SCC (*p* > 0.05).

**TABLE 2:** Differences in PD‐L1 protein expression in different pathological types of lung cancer.

Groups		AC (*n* = 21)	SCC (*n* = 14)	*p*
Threshold of 1%	PD‐L1 negative (< 1%)	7 (50.0%)	7 (50.0%)	0.324
	PD‐L1 positive (≥ 1%)	14 (66.7%)	7 (33.3%)	
Threshold of 50%	Weak PD‐L1 expression (< 50%)	7 (70.0%)	3 (30.0%)	0.704
	Strong PD‐L1 expression (≥ 50%)	14 (56.0%)	11 (44.0%)	
Thresholds of 1% and 50%	PD‐L1 nonexpression (< 1%)	7 (50.0%)	7 (50.0%)	0.683
	Low expression (1%–49%)	7 (63.6.1%)	4 (36.4%)	
	High expression (≥ 50%)	7 (70.0%)	3 (30.0%)	

*Note:* AC, adenocarcinoma; SCC, squamous carcinoma.

The IHC results showed PD‐L1 < 1% in 14 cases, PD‐L1 1 to 49% in 11 cases, and PD‐L1 ≥ 50% in 10 cases. Age, sex, body mass index (BMI), maximum tumor diameter (measured by T2WI), tumor stage, lesion location, lesion site, and smoking status were not statistically different between the different PD‐L1 expression groups (Tables 3, 4, and 5).

**TABLE 3:** Differences in clinical and imaging data of patients between PD‐L1‐negative and PD‐L1‐positive groups (threshold of 1%).

	PD‐L1‐negative (*n* = 14)	PD‐L1‐positive (*n* = 21)	*p*
Age (years)	67.071 ± 9.739	67.048 ± 9.041	0.994
BMI (kg/m^2^)	21.340 ± 2.829	22.521 ± 2.451	0.198
Gender (*n*, %)			0.133
Male	14 (45.2%)	17 (54.8%)	
Female	0 (0.0%)	4 (100.0%)	
Maximum diameter of tumor (mm)	36.550 (31.000, 62.750)	38.500 (31.000, 58.500)	0.960
ADC means (×10^−6^ mm^2^/s)	1.191 ± 0.234	1.349 ± 0.209	0.052
Stage of tumor (*n*, %)			1.000
I–II	4 (44.4%)	5 (55.6%)	
III–IV	10 (38.5%)	16 (61.5%)	
Location of lesion (*n*, %)			0.486
Right	9 (45.0%)	11 (55.0%)	
Left	5 (33.3%)	10 (66.7%)	
Site of lesion (*n*, %)			1.000
Upper and middle lobes	10 (41.7%)	14 (58.3%)	
Lower lobe	4 (36.4%)	7 (63.6%)	
Smoking (*n*, %)			0.486
Yes	9 (45.0%)	11 (55.0%)	
No	5 (33.3%)	10 (66.7%)	

Abbreviation: BMI, body mass index. Maximum tumor diameter measured on T2WI.

**TABLE 4:** Differences in clinical and imaging data of patients between weak and strong PD‐L1 expression groups (threshold of 50%).

	Weak PD‐L1 expression (*n* = 10)	Strong PD‐L1 expression (*n* = 25)	*p*
Age (years)	69.500 (66.750,73.500)	66.400 ± 9.853	0.635
BMI (kg/m^2^)	22.109 ± 2.713	22.024 ± 2.658	0.934
Gender (*n*, %)			0.061
Male	7 (22.6%)	24 (77.4%)	
Female	3 (75.0%)	1 (25.0%)	
Maximum diameter of tumor (mm)	50.040 ± 26.153	37.200 (31.350, 56.000)	0.453
ADC means (×10^−6^ mm^2^/s)	1.298 ± 0.205	1.281 ± 0.243	0.844
Stage of tumor (*n*, %)			0.694
I‐II	3 (33.3%)	6 (66.7%)	
III‐IV	7 (26.9%)	19 (73.1%)	
Location of lesion (*n*, %)			0.266
Right	4 (20.0%)	16 (80.0%)	
Left	6 (40.0%)	9 (60.0%)	
Site of lesion (*n*, %)			0.689
Upper and middle lobes	6 (25.0%)	18 (75.0%)	
Lower lobe	4 (36.4%)	7 (63.6%)	
Smoking (*n*, %)			0.712
Yes	5 (25.0%)	15 (75.0%)	
No	5 (33.3%)	10 (66.7%)	

Abbreviation: BMI, body mass index. Maximum tumor diameter measured on T2WI.

**TABLE 5:** Differences in clinical and imaging data of patients among PD‐L1 nonexpression, low expression, and high expression groups (threshold of 1% and 50%).

	PD‐L1 nonexpression (*n* = 14)	Low PD‐L1 expression (*n* = 11)	High PD‐L1 expression (*n* = 10)	*p*
Age (years)	67.071 ± 9.739	65.545 ± 10.405	69.500 (66.750, 73.500)	0.635
BMI (kg/m^2^)	21.340 ± 2.829	22.896 ± 2.250	22.109 ± 2.713	0.351
Gender (n, %)				0.055
Male	14 (45.2%)	10 (32.3%)	7 (22.6%)	
Female	0 (0.0%)	1 (25.0%)	3 (75.0%)	
Maximum diameter of tumor (mm)	36.550 (31.000, 62.750)	41.146 ± 16.394	50.040 ± 26.153	0.453
ADC means (×10^−6^ mm^2^/s)	1.191 ± 0.234	1.394 ± 0.212	1.298 ± 0.205	0.084
Stage of tumor (*n*, %)				0.793
I–II	4 (44.4%)	2 (22.2%)	3 (33.3%)	
III–IV	10 (38.5%)	9 (34.6%)	7 (26.9%)	
Location of lesion (*n*, %)				0.475
Right	9 (45.0%)	7 (35.0%)	4 (20.0%)	
Left	5 (33.3%)	4 (26.7%)	6 (40.0%)	
Site of lesion (*n*, %)				0.810
Upper and middle lobes	10 (41.7%)	8 (33.3%)	6 (25.0%)	
Lower lobe	4 (36.4%)	3 (27.3%)	4 (36.4%)	
Smoking (*n*, %)				0.760
Yes	9 (45.0%)	6 (30.0%)	5 (25.0%)	
No	5 (33.3%)	5 (33.3%)	5 (33.3%)	

Abbreviation: BMI, body mass index. Maximum tumor diameter measured on T2WI.

### 3.2 Differences in ADC Means Between PD‐L1 Expression Clusters by Different Thresholds

A 1% threshold revealed no statistically significant difference between the ADC means of the PD‐L1‐negative and PD‐L1‐positive groups (*p* = 0.052, Table 6). The mean ADC value for the PD‐L1 negative group was (1.191 ± 0.234) × 10^−6^ mm^2^/s, whereas the mean ADC value for the PD‐L1 positive group was (1.349 ± 0.209) × 10^−6^ mm^2^/s.

**TABLE 6:** Differences in ADC means between PD‐L1 expression groups with different threshold classifications.

Groups		ADC mean value (×10^−6^ mm^2^/s)	*p*
Threshold of 1%	PD‐L1‐negative (< 1%)	1.191 ± 0.234	0.052
	PD‐L1‐positive (≥ 1%)	1.349 ± 0.209	
Threshold of 50%	Weak PD‐L1 expression (< 50%)	1.298 ± 0.205	0.844
	Strong PD‐L1 expression (≥ 50%)	1.281 ± 0.243	
Thresholds of 1% and 50%	PD‐L1 nonexpression (< 1%)	1.191 ± 0.234	0.084
	Low PD‐L1 expression (1%–49%)	1.394 ± 0.212	
	High PD‐L1 expression (≥ 50%)	1.298 ± 0.243	

Abbreviation: ADC, apparent diffusion coefficient.

A 50% threshold revealed no statistically significant difference in ADC means between the weak and strong PD‐L1 expression groups (*p* = 0.844, Table 6). The mean ADC values for the weak PD‐L1 expression group were (1.298 ± 0.205) × 10^−6^ mm^2^/s, whereas those for the strong PD‐L1 expression group were (1.281 ± 0.243) × 10^−6^ mm^2^/s.

The application of 1% and 50% as thresholds revealed no statistically significant differences in the ADC means between the PD‐L1 nonexpression, low expression, and high expression groups (*p* = 0.084, Table 6). Nevertheless, a two‐way LSD comparison revealed a statistically significant difference between the PD‐L1 nonexpression and low expression groups (*p* = 0.028). The mean ADC value was (1.191 ± 0.234) × 10^−6^ mm^2^/s in the PD‐L1 nonexpression group, (1.394 ± 0.212) × 10^−6^ mm^2^/s in the PD‐L1 low expression group, and (1.394 ± 0.212) × 10^−6^ mm^2^/s in the PD‐L1 high expression group.

### 3.3 DCE‐MRI Quantitative Perfusion Histogram Parameters Predicting PD‐L1 Expression for Different Threshold Classifications

#### 3.3.1 DCE‐MRI Quantitative Perfusion Histogram Parameters for Predicting Threshold 1% PD‐L1 Expression

A threshold of 1% was employed to dichotomize the patient cohort into those exhibiting PD‐L1‐negative and PD‐L1‐positive groups. No statistically significant differences were observed between the two groups for the nine perfusion histogram parameters (*K* ^trans^, *K*_ep_, *V*_e_, *V*_p_, and *F*_p_ for ECM and *K* ^trans^, *K*_ep_, *V*_p_, and *V*_p_ for ETM) or for the histogram parameters between the two groups (*p* > 0.05). Details are provided in the Tables S1.1–S1.9 (see ESM).

#### 3.3.2 DCE‐MRI Quantitative Perfusion Histogram Parameters for Predicting Threshold 50% PD‐L1 Expression

**FIGURE 3:**
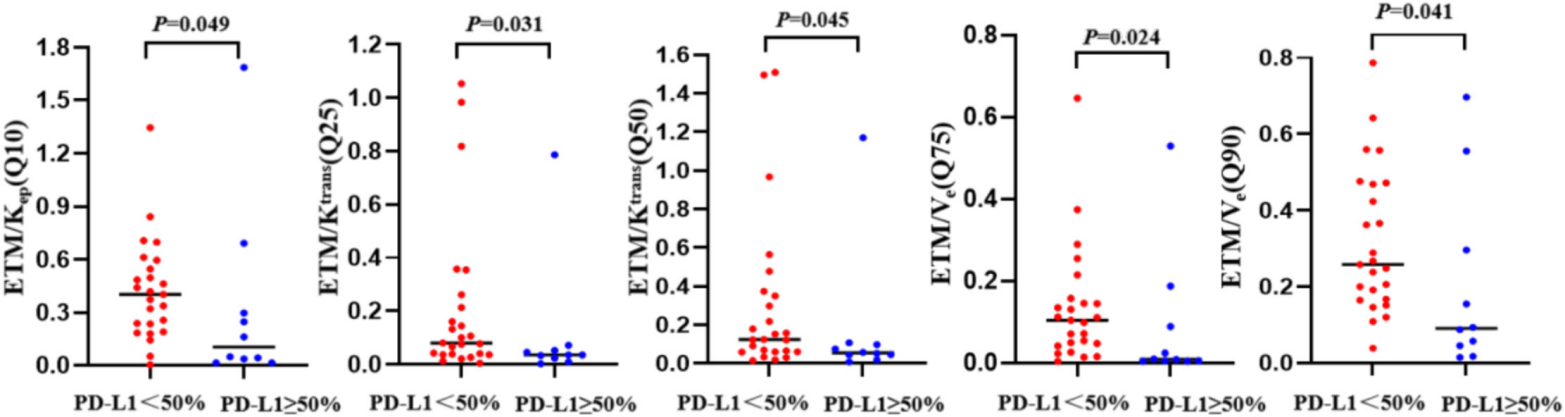
Scatter plot of quantitative perfusion histogram parameters for predicted PD‐L1 expression (threshold at 50%). *V*_e_ (*Q*75 and *Q*90) is from ECM; other parameters are from ETM. The short black horizontal line represents the median.

**FIGURE 4:**
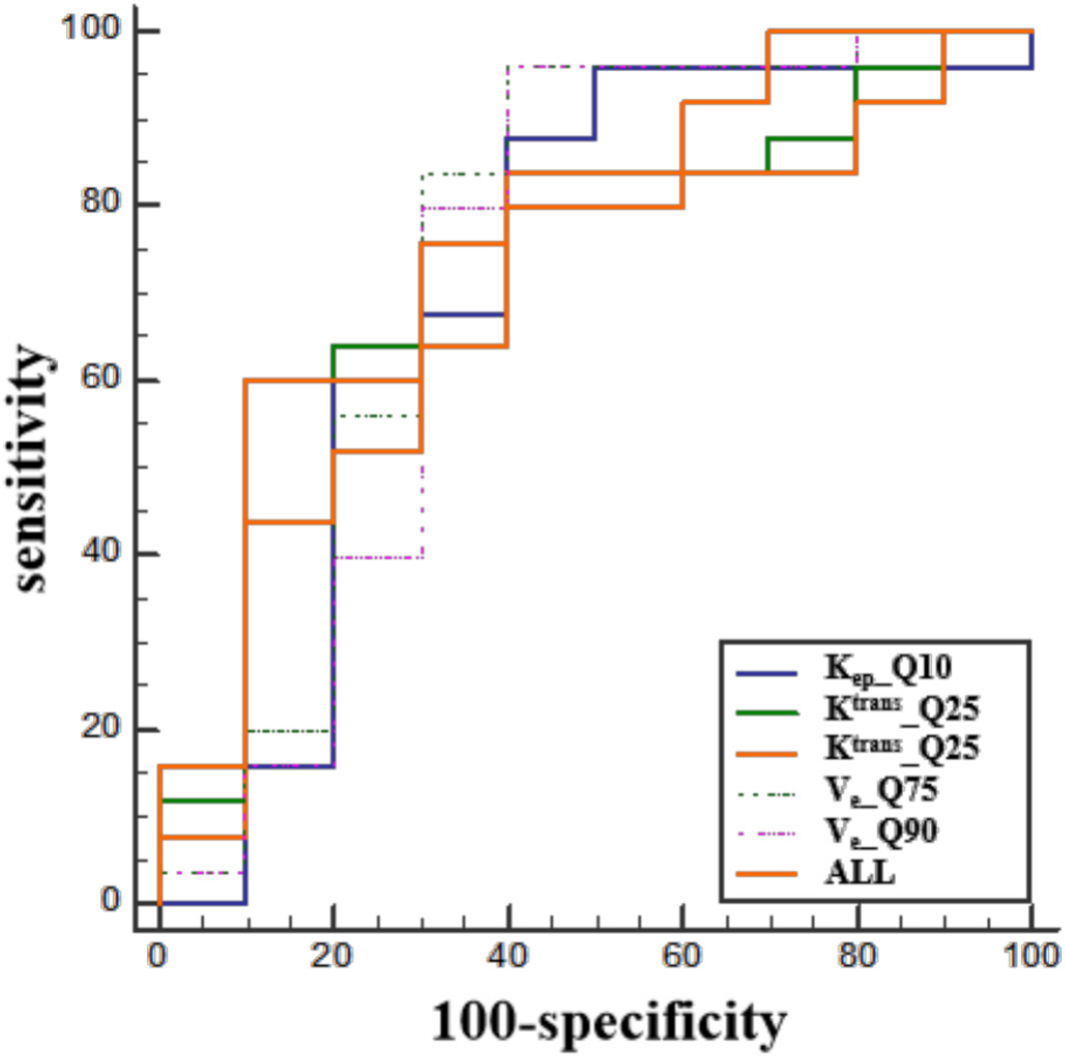
ROC curves for DCE‐MRI perfusion histogram parameters predicting 50% PD‐L1 expression at threshold. ETM/*K*_ep_ *Q*10 (AUC = 0.716, 95% CI 0.539–0.855). ETM/*K* ^trans^ Q25 (AUC = 0.736, 95% CI 0.560–0.870), *Q*50 (AUC = 0.720, 95% CI 0.543–0.858). ECM/*V*_e_ *Q*75 (AUC = 0.748, 95% CI 0.573–0.879), *Q*90 (AUC = 0.724, 95% CI 0.547–0.861). AUC = 0.740 (95% CI 0.564–0.873) for all perfusion histogram parameters.

A 50% cut‐off was employed to categorize patients according to their weak or strong PD‐L expression. Statistical differences were observed for *Q*10 (*p* = 0.049) for ETM/*K*_ep_, *Q*25 (*p* = 0.031), and *Q*50 (*p* = 0.045) for ETM/*K* ^trans^ and *Q*75 (*p* = 0.024) and *Q*90 (*p* = 0.041) for ECM/*V*_e_ between the two groups. The results demonstrated that ETM/*K*_ep_ (*Q*10), ETM/*K* ^trans^ (*Q*25 and *Q*50), and ECM/*V*_e_ (*Q*75 and *Q*90) were elevated in the weak PD‐L1 expression group relative to the strong PD‐L1 expression group (Figure 3). The AUCs of the aforementioned histogram parameters predicting PD‐L1 expression with a threshold of 50% were 0.716, 0.736, 0.720, 0.748, and 0.724, respectively. The combined AUC of all parameters was 0.740, with a sensitivity of 0.760 and a specificity of 0.700 (Figure 4). As illustrated in Tables S2.1–S2.9 (see ESM), no statistically significant differences were observed in the remaining parameters between the two groups (*p* > 0.05).

#### 3.3.3 DCE‐MRI Quantitative Perfusion Histogram Parameters for Predicting Threshold 1% and 50% PD‐L1 Expression

**FIGURE 5:**
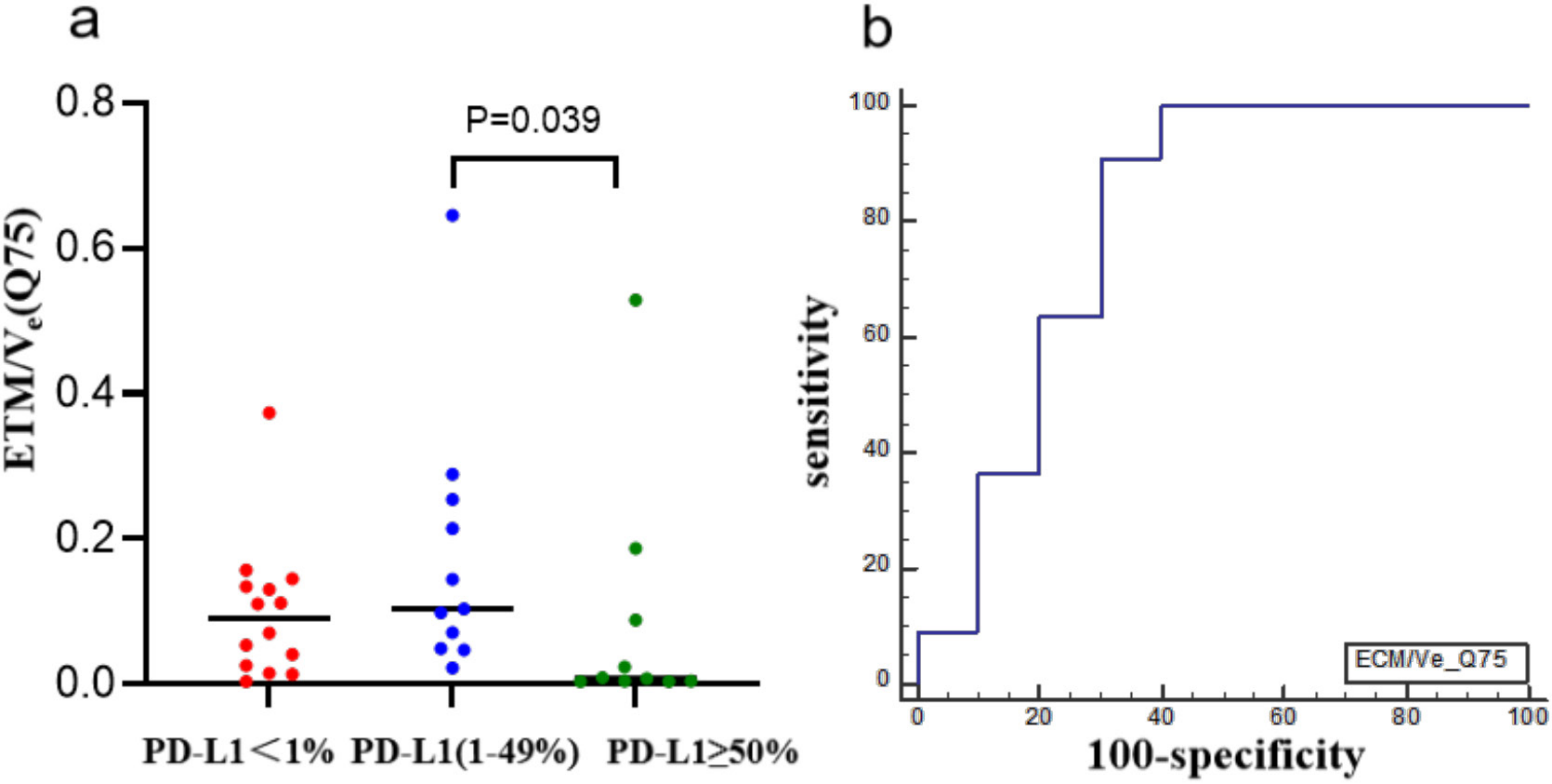
(a) Scatter plot of ECM/*V*_e_ (*Q*75) in the three classified PD‐L1 expression groups. This parameter was statistically different among the PD‐L1 nonexpression, low expression, and high expression groups (*p* = 0.044). In the pairwise comparison, a statistically different parameter was found between the PD‐L1 low and high expression groups (*p* = 0.039), and its value was significantly higher in the PD‐L1 low than in the PD‐L1 high expression group. The short black horizontal line represents the median. (b) ROC curves of ECM/*V*_e_ (*Q*75) predicting PD‐L1 low and high expression, the AUC was 0.800 (95% CI 0.570–0.940), and the sensitivity and specificity were 0.909 and 0.700, respectively.

The patients were divided into three groups based on the thresholds of 1% and 50%, which were used to categorize their PD‐L1 expression levels as nonexpression, low expression, and high expression. The K‐W test demonstrated that there was a statistically significant difference in the distribution of ECM/*V*_e_ (*Q*75) among the three groups (*p* = 0.044). In a two‐way comparison, the parameter exhibited a statistically significant difference between low and high PD‐L1 expression (*p* = 0.039). Furthermore, its ability to predict low and high PD‐L1 expression was demonstrated by an AUC of 0.800 (95% CI 0.570–0.940), sensitivity of 0.909, and specificity of 0.700 (Figure 5). No statistically significant differences were observed in the remaining parameters between the three groups (*p* > 0.05). The specific data were presented in the Tables S3.1–S3.9 (see ESM).

## 4 Discussion

The assessment of PD‐L1 expression may facilitate the development of personalized treatment plans as a consequence of the creation of PD‐1 and PD‐L1 inhibitors. Noninvasive imaging has been extensively employed in the evaluation of pathological staging, efficacy, and prognosis in lung cancer, and is anticipated to be utilized for the assessment of immune pathway protein expression [21]. In this study, we extracted hemodynamic parameters and their histogram parameters using a DCE‐MRI dual blood supply model based on DCE‐MRI and found that these parameters could predict the PD‐L1 expression scoring system at different thresholds in patients with NSCLC. The ETM/*K*_ep_ (*Q*10), ETM/*K* ^trans^ (*Q*25 and *Q*50), and ECM/*V*_e_ (*Q*75 and *Q*90) parameters were used to stratify PD‐L1 thresholds for 50% of NSCLC, whereas the ECM/*V*_e_ (*Q*75) parameter was able to discriminate between those with PD‐L1 levels of 1%–49% and those with PD‐L1 levels of ≥ 50%.

Prior studies have demonstrated that DCE‐MRI imaging parameters are indicative of tumor biology, encompassing aspects such as vascularization, neoangiogenesis, and proliferative potential [22, 23]. *K* ^trans^ and *K*_ep_ were found to be associated with several key parameters of microcirculatory blood flow, including blood flow velocity, vessel wall exchange area, and vessel wall permeability [24]. A moderate correlation was identified between the relevant parameters of *K* ^trans^, *K*_ep_, and PD‐L1 expression [25]. In their respective studies, Meyer et al. and Tekiki et al. employed DCE‐MRI to assess SCC of the head and neck and oral cavity, respectively. Their findings indicated a correlation between increasing blood perfusion levels and elevated PD‐L1 expression [26, 27]. In a study of lung cancer, Wen et al. [28] proposed a correlation between PD‐L1 expression in non–small cell lung cancer and vascular convergence. Furthermore, it has been proposed that elevated PD‐L1 expression facilitates tumor progression, necessitating a greater blood supply to transport adequate nutrients [29]. Consequently, tumors with high PD‐L1 expression are likely to exhibit elevated *K* ^trans^ and *K*_ep_ values, a finding that is corroborated by the results of this study. The histogram parameters serve to visualize the microstructure of tumor components [30], whereas the percentile of the histogram describes the concentration trend of the data [29]. In this context, *Q*50 is situated in the middle of the histogram and represents the median. Meanwhile, *Q*10 and *Q*25 are located on the left side of the histogram, representing smaller data, whereas *Q*75 and *Q*90 are situated on the right side of the histogram, representing larger data. The findings revealed that tumors with low PD‐L1 expression exhibited higher *V*_e_ values, indicating a larger extracellular gap than tumors with high PD‐L1 expression. The aforementioned results can be interpreted as follows: tumors with a high level of PD‐L1 expression exhibit substantial infiltration of immune cells or cell proliferation and tumor neovascularization, as evidenced by low *V*_e_ values and high *K* ^trans^ and *K*_ep_ values.

This study did not identify any notable discrepancies. No significant differences in perfusion parameters between the dichotomous PD‐L1 expression groups with a threshold of 1% were found. It was postulated that the utilization of a threshold value of 50% may have resulted in an augmented divergence in the parameter values between the groups. The threshold for positive PD‐L1 expression is variable, with options including 1%, 5%, 10%, 20%, 25%, and 50%. Most studies have employed a threshold of 1% or 50%. Advanced NSCLC with TPS not less than 50% or 1% has become one of the criteria for the use of PD‐L1 immunosuppressants. The results showed that the AUC of PD‐L1 prediction models with a threshold of 50% was generally higher than those with a threshold of 1% [31, 32, 33]. This suggests that the difference in imaging characteristics between the PD‐L1 expression ≥ 50% and < 50% groups may be greater than that between the PD‐L1 expression ≥ 1% and < 1% groups.

The present study did not find an association between ADC values and PD‐L1 expression in NSCLC. Prior research has indicated that ADC values are reflective of tumor microstructure [34, 35]. In a study of lung cancer, D (diffusion coefficient) was observed to be lower in the PD‐L1‐positive group than in the PD‐L1‐negative group [36]. Bortolotto et al. reported a positive correlation between D* values (diffusion coefficients associated with perfusion) and PD‐L1‐positive expression but found no correlation between D and PD‐L1 expression [37]. The level of ADC is related to a number of factors, including (1) ADC values are influenced by the EES. Tumors that express high levels of PD‐L1 have a large number of immune cells infiltrating the interstitium [38]. This results in a limitation of the diffusion of water molecules in the interstitial space of the cells, which manifests as low ADC values. (2) Increased ADC is associated with tumor necrosis [39, 40]. One study demonstrated that AC with cysts was significantly associated with the expression of PD‐L1 [41]. Furthermore, tumors with high PD‐L1 expression have high vascular invasiveness [29], which results in lesion ischemia and hypoxia and cystic necrosis. The negative results observed in this study may be related to the small sample size, which requires expansion to facilitate further exploration.

It should be noted that the present study is subject to several limitations. Firstly, the study was conducted at a single center and lacked external validation. Furthermore, the sample size was limited, and the different pathological types of lung cancer were not analyzed separately. These shortcomings will be addressed in future studies with larger sample sizes. Secondly, accurately matching surgical specimens to MRI images is a challenging endeavor. Subsequently, the pathologist will select three to five successive slices, centered on the largest section of disease visible in the image, in order to provide maximum coverage of the area of interest.

## 5 Conclusion

DCE‐MRI quantitative analysis is a valuable tool for the evaluation of PD‐L1 expression in patients with NSCLC. It offers a noninvasive and reproducible methodology for the assessment of cellular pathways, which can be employed to assist in the stratification of PD‐L1 expression in NSCLC patients and the development of accurate treatment plans.

## Author Contributions

Z.Z. conceived the idea and participated in the design, data collection, data analysis, and interpretation of the data and developed and reviewed the manuscript. C.Y. conceived the idea and participated in the design, data collection, data analysis, and interpretation of the data and developed and reviewed the manuscript. F.Z. participated in data analysis and interpretation of the data. Q.L. participated in the interpretation of the data and reviewed the manuscript. C.S. reviewed the manuscript. H.J. participated in the design and data collection, conducted laboratory procedures, and reviewed the manuscript.

## Ethics Statement

Ethical approval was obtained for this study (Approval No. 202063).

## Consent

Each patient provided informed consent for the MRI examination.

## Conflicts of Interest

The authors declare no conflicts of interest.

## Supporting information


**Table S1:1** Differences of ECM *F*_p_ perfusion histogram parameters between PD‐L1‐negative and PD‐L1‐positive expression groups.
**Table S1:**2 Differences of ECM Kep perfusion histogram parameters between PD‐L1‐negative and PD‐L1‐positive expression groups.
**Table S1:**3 Differences of ECM Ktrans perfusion histogram parameters between PD‐L1‐negative and PD‐L1‐positive expression groups.
**Table S1:**4 Differences of ECM Ve perfusion histogram parameters between PD‐L1‐negative and PD‐L1‐positive expression groups.
**Table S1:**5 Differences of ECM Vp perfusion histogram parameters between PD‐L1‐negative and PD‐L1‐positive expression groups.
**Table S1:**6 Differences of ETM Ktrans perfusion histogram parameters between PD‐L1‐negative and PD‐L1‐positive expression groups.
**Table S1:**7 Differences of ETM Kep perfusion histogram parameters between PD‐L1‐negative and PD‐L1‐positive expression groups.
**Table S1:**8 Differences of ETM Ve perfusion histogram parameters between PD‐L1‐negative and PD‐L1‐positive expression groups.
**Table S1:**9 Differences of ETM Vp perfusion histogram parameters between PD‐L1‐negative and PD‐L1‐positive expression groups.
**Table S2:**1 Differences in ECM Fp perfusion histogram parameters between groups with weak and strong PD‐L1 expressions.
**Table S2:**2 Differences in ECM Kep perfusion histogram parameters between groups with weak and strong PD‐L1 expressions.
**Table S2:**3 Differences in ECM Ktrans perfusion histogram parameters between groups with weak and strong PD‐L1 expressions.
**Table S2:**4 Differences in ECM Ve perfusion histogram parameters between groups with weak and strong PD‐L1 expressions.
**Table S2:**5 Differences in ECM Vp perfusion histogram parameters between groups with weak and strong PD‐L1 expressions.
**Table S2:**6 Differences in ETM Ktrans perfusion histogram parameters between groups with weak and strong PD‐L1 expressions.
**Table S2:**7 Differences in ETM Kep perfusion histogram parameters between groups with weak and strong PD‐L1 expressions.
**Table S2:**8 Differences in ETM Ve perfusion histogram parameters between groups with weak and strong PD‐L1 expressions.
**Table S2:**9 Differences in ETM Vp perfusion histogram parameters between groups with weak and strong PD‐L1 expressions.
**Table S3:**1 Differences in perfusion histogram parameters of ECM Fp between PD‐L1 nonexpression, low expression, and high expression groups.
**Table S3:**2 Differences in perfusion histogram parameters of ECM Kep between PD‐L1 nonexpression, low expression, and high expression groups.
**Table S3:**3 Differences in perfusion histogram parameters of ECM Ktrans between PD‐L1 nonexpression, low expression, and high expression groups.
**Table S3:**4 Differences in perfusion histogram parameters of ECM Ve between PD‐L1 nonexpression, low expression, and high expression groups.
**Table S3:**5 Differences in perfusion histogram parameters of ECM Vp between PD‐L1 nonexpression, low expression, and high expression groups.
**Table S3:**6 Differences in perfusion histogram parameters of ETM Ktrans between PD‐L1 nonexpression, low expression, and high expression groups.
**Table S3:**7 Differences in perfusion histogram parameters of ETM Kep between PD‐L1 nonexpression, low expression, and high expression groups.
**Table S3:**8 Differences in perfusion histogram parameters of ETM Ve between PD‐L1 nonexpression, low expression, and high expression groups.
**Table S3:**9 Differences in perfusion histogram parameters of ETM Vp between PD‐L1 nonexpression, low expression, and high expression groups.

## Data Availability

The data that support the findings of this study are available from the corresponding author upon reasonable request.
