# Supplementary material for: Evaluation of PD‐L1 Expression in Patients With Non–Small Cell Lung Cancer Using DCE‐MRI Quantitative Analysis
Source: Clin Respir J. 2025 Sep 27;19(10):e70125. doi: 10.1111/crj.70125 (PMC12475933; doi:10.1111/crj.70125)
Supplement: Supplementary file 1 — Table S1:1 Differences of ECM F p perfusion histogram parameters between PD‐L1‐negative and PD‐L1‐positive expression groups. Table S1:2 Differences of ECM Kep perfusion histogram parameters between PD‐L1‐negative and PD‐L1‐positive expression groups. Table S1:3 Differences of ECM Ktrans perfusion histogram parameters between PD‐L1‐negative and PD‐L1‐positive expression groups. Table S1:4 Differences of ECM Ve perfusion histogram parameters between PD‐L1‐negative and PD‐L1‐positive expression groups. Table S1:5 Differences of ECM Vp perfusion histogram parameters between PD‐L1‐negative and PD‐L1‐positive expression groups. Table S1:6 Differences of ETM Ktrans perfusion histogram parameters between PD‐L1‐negative and PD‐L1‐positive expression groups. Table S1:7 Differences of ETM Kep perfusion histogram parameters between PD‐L1‐negative and PD‐L1‐positive expression groups. Table S1:8 Differences of ETM Ve perfusion histogram parameters between PD‐L1‐negative and PD‐L1‐positive expression groups. Table S1:9 Differences of ETM Vp perfusion histogram parameters between PD‐L1‐negative and PD‐L1‐positive expression groups. Table S2:1 Differences in ECM Fp perfusion histogram parameters between groups with weak and strong PD‐L1 expressions. Table S2:2 Differences in ECM Kep perfusion histogram parameters between groups with weak and strong PD‐L1 expressions. Table S2:3 Differences in ECM Ktrans perfusion histogram parameters between groups with weak and strong PD‐L1 expressions. Table S2:4 Differences in ECM Ve perfusion histogram parameters between groups with weak and strong PD‐L1 expressions. Table S2:5 Differences in ECM Vp perfusion histogram parameters between groups with weak and strong PD‐L1 expressions. Table S2:6 Differences in ETM Ktrans perfusion histogram parameters between groups with weak and strong PD‐L1 expressions. Table S2:7 Differences in ETM Kep perfusion histogram parameters between groups with weak and strong PD‐L1 expressions. Table S2:8 Difference [file CRJ-19-e70125-s001.docx]

**Supplementary table 1.1 Differences of ECM F_p_ perfusion histogram parameters between PD-L1 negative and positive expression groups**

| **Parameters** | **PD-L1 negative (n=14)** | **PD-L1 positive (n=21)** | ***P* value** |
| --- | --- | --- | --- |
| Meanvalue | 0.145 (0.076,0.595) | 0.113 (0.062,0.217) | 0.329 |
| Skewness | 1.257±1.220 | 0.876 (0.547,2.198) | 1.000 |
| Kurtosis | 2.295 (-0.455,8.050) | 0.754 (0.062,6.144) | 0.946 |
| Uniformity | 0.461±0.098 | 0.474 (0.331,0.525) | 0.614 |
| Energy | 0.011 (0.006,0.017) | 0.009 (0.008,0.014) | 0.973 |
| Entropy | 6.773±0.785 | 7.069 (6.422,7.200) | 0.946 |
| Quantity5 | 0.030 (0.017,0.172) | 0.031 (0.019,0.065) | 0.501 |
| Quantity10 | 0.052 (0.027,0.241) | 0.038 (0.027,0.094) | 0.439 |
| Quantity25 | 0.086 (0.045,0.388) | 0.072 (0.038,0.147) | 0.297 |
| Quantity50 | 0.127 (0.074,0.541) | 0.106 (0.057,0.200) | 0.266 |
| Quantity75 | 0.181 (0.103,0.730) | 0.147 (0.075,0.272) | 0.266 |
| Quantity90 | 0.259 (0.128,0.994) | 0.189 (0.102,0.385) | 0.419 |
| Quantity95 | 0.321 (0.146,1.159) | 0.209 (0.119,0.464) | 0.400 |

**Supplementary table 1.2 Differences of ECM K_ep_ perfusion histogram parameters between PD-L1 negative and positive expression groups**

| **Parameters** | **PD-L1 negative (n=14)** | **PD-L1 positive (n=21)** | ***P* value** |
| --- | --- | --- | --- |
| Meanvalue | 4.929 (4.040,5.181) | 3.992±1.704 | 0.178 |
| Skewness | -0.367 (-0.512,0.043) | 0.118 (-0.527,0.702) | 0.167 |
| Kurtosis | -1.735 (-1.870,-1.586) | -1.590 (-1.890,-0.469) | 0.266 |
| Uniformity | 0.251 (0.054,0.320) | -0.001 (-0.261,0.331) | 0.225 |
| Energy | 0.255±0.163 | 0.305±0.186 | 0.420 |
| Entropy | 4.363±1.279 | 3.954±1.360 | 0.380 |
| Quantity5 | 0.003 (0.002,0.005) | 0.003 (0.002,0.004) | 0.522 |
| Quantity10 | 0.007 (0.005,0.010) | 0.006 (0.005,0.009) | 0.522 |
| Quantity25 | 0.017 (0.012,0.024) | 0.014 (0.012,0.022) | 0.480 |
| Quantity50 | 0.204 (0.024,0.581) | 0.028 (0.024,0.621) | 0.501 |
| Quantity75 | 1.150±0.781 | 0.668 (0.320,1.433) | 0.297 |
| Quantity90 | 2.747±1.508 | 1.738 (1.013,3.130) | 0.329 |
| Quantity95 | 4.111±1.787 | 3.535±1.778 | 0.356 |

**Supplementary table 1.3 Differences of ECM K^trans^ perfusion histogram parameters between PD-L1 negative and positive expression groups**

| **Parameters** | **PD-L1 negative (n=14)** | **PD-L1 positive (n=21)** | ***P* value** |
| --- | --- | --- | --- |
| Meanvalue | 0.178 (0.094,0.624) | 0.119 (0.069,0.315) | 0.312 |
| Skewness | 5.388±4.820 | 6.884 (3.674,11.372) | 0.201 |
| Kurtosis | 63.217 (0.245,123.217) | 96.139 (36.038,250.804) | 0.167 |
| Uniformity | 0.329 (-0.272,0.513) | 0.150 (-0.223,0.424) | 0.501 |
| Energy | 0.053±0.044 | 0.045 (0.029,0.082) | 0.459 |
| Entropy | 5.330±1.390 | 4.693±1.199 | 0.225 |
| Quantity5 | 0.032 (0.021,0.204) | 0.030 (0.024,0.074) | 0.762 |
| Quantity10 | 0.056 (0.031,0.276) | 0.050 (0.029,0.105) | 0.439 |
| Quantity25 | 0.092 (0.052,0.420) | 0.076 (0.045,0.163) | 0.346 |
| Quantity50 | 0.135 (0.085,0.568) | 0.115 (0.061,0.233) | 0.329 |
| Quantity75 | 0.205 (0.113,0.755) | 0.152 (0.082,0.370) | 0.281 |
| Quantity90 | 0.284 (0.148,1.021) | 0.202 (0.108,0.541) | 0.312 |
| Quantity95 | 0.410 (0.179,1.192) | 0.258 (0.130,0.632) | 0.312 |

**Supplementary table 1.4 Differences of ECM V_e_ perfusion histogram parameters between PD-L1 negative and positive expression groups**

| **Parameters** | **PD-L1 negative (n=14)** | **PD-L1 positive (n=21)** | ***P* value** |
| --- | --- | --- | --- |
| Meanvalue | 0.349±0.127 | 0.400±0.201 | 0.408 |
| Skewness | 0.836±0.670 | 0.369 (-0.100,1.700) | 0.206 |
| Kurtosis | -1.293 (-1.641,0.048) | -1.690 (-1.807,1.164) | 0.577 |
| Uniformity | -0.327 (-0.619,0.006) | -0.083 (-0.848,0.173) | 0.308 |
| Energy | 0.129 (0.033,0.281) | 0.202 (0.067,0.407) | 0.596 |
| Entropy | 4.443±1.456 | 4.011±1.709 | 0.444 |
| Quantity5 | 0.001 (0.000,0.008) | 0.001 (0.000,0.001) | 0.654 |
| Quantity10 | 0.001 (0.001,0.016) | 0.002 (0.001,0.003) | 0.654 |
| Quantity25 | 0.004 (0.002,0.030) | 0.004 (0.002,0.007) | 0.817 |
| Quantity50 | 0.009 (0.005,0.048) | 0.007 (0.003,0.025) | 0.714 |
| Quantity75 | 0.090 (0.023,0.137) | 0.071 (0.008,0.201) | 0.946 |
| Quantity90 | 0.272±0.170 | 0.238 (0.091,0.475) | 0.903 |
| Quantity95 | 0.413±0.198 | 0.431±0.261 | 0.825 |

**Supplementary table 1.5 Differences of ECM V_p_ perfusion histogram parameters between PD-L1 negative and positive expression groups**

| **Parameters** | **PD-L1 negative (n=14)** | **PD-L1 positive (n=21)** | ***P* value** |
| --- | --- | --- | --- |
| Meanvalue | 0.114 (0.088,0.373) | 0.118 (0.062,0.272) | 0.522 |
| Skewness | 1.217 (0.138,4.433) | 0.741 (0.231,2.806) | 0.788 |
| Kurtosis | 4.305 (0.015,29.960) | 0.988 (-0.210,14.766) | 0.400 |
| Uniformity | 0.392 (-0.193,0.577) | 0.335 (0.072,0.494) | 0.501 |
| Energy | 0.012 (0.007,0.031) | 0.011 (0.007,0.025) | 0.762 |
| Entropy | 6.432±1.095 | 6.882 (5.718,7.325) | 0.920 |
| Quantity5 | 0.018 (0.002,0.061) | 0.005 (0.002,0.029) | 0.266 |
| Quantity10 | 0.033 (0.007,0.130) | 0.016 (0.005,0.048) | 0.201 |
| Quantity25 | 0.063 (0.035,0.262) | 0.065 (0.019,0.128) | 0.346 |
| Quantity50 | 0.102 (0.078,0.369) | 0.100 (0.043,0.222) | 0.459 |
| Quantity75 | 0.146 (0.113,0.451) | 0.134 (0.065,0.328) | 0.363 |
| Quantity90 | 0.199 (0.148,0.531) | 0.163 (0.104,0.414) | 0.329 |
| Quantity95 | 0.243 (0.168,0.591) | 0.188 (0.114,0.473) | 0.297 |

**Supplementary table 1.6 Differences of ETM K^trans^ perfusion histogram parameters between PD-L1 negative and positive expression groups**

| **Parameters** | **PD-L1 negative (n=14)** | **PD-L1 positive (n=21)** | ***P* value** |
| --- | --- | --- | --- |
| Meanvalue | 0.145 (0.065,0.752) | 0.101 (0.054,0.186) | 0.189 |
| Skewness | 1.167 (0.539,3.117) | 1.085 (0.522,2.388) | 0.840 |
| Kurtosis | 3.347 (-0.204,21.098) | 1.440 (0.154,12.976) | 0.973 |
| Uniformity | 0.411 (0.343,0.520) | 0.438 (0.225,0.490) | 0.711 |
| Energy | 0.019±0.012 | 0.016 (0.008,0.022) | 0.567 |
| Entropy | 6.332±0.888 | 6.127 (5.738,7.230) | 0.686 |
| Quantity5 | 0.029 (0.014,0.150) | 0.021 (0.009,0.047) | 0.213 |
| Quantity10 | 0.044 (0.020,0.0255) | 0.030 (0.015,0.072) | 0.239 |
| Quantity25 | 0.079 (0.038,0.473) | 0.053 (0.029,0.119) | 0.148 |
| Quantity50 | 0.124 (0.061,0.665) | 0.092 (0.046,0.169) | 0.178 |
| Quantity75 | 0.193 (0.089,0.901) | 0.130 (0.067,0.240) | 0.201 |
| Quantity90 | 0.266 (0.108,1.245) | 0.163 (0.091,0.329) | 0.281 |
| Quantity95 | 0.320 (0.122,1.532) | 0.187 (0.108,0.402) | 0.312 |

**Supplementary table 1.7 Differences of ETM K_ep_ perfusion histogram parameters between PD-L1 negative and positive expression groups**

| **Parameters** | **PD-L1 negative (n=14)** | **PD-L1 positive (n=21)** | ***P* value** |
| --- | --- | --- | --- |
| Meanvalue | 1.152±0.714 | 0.982 (0.561,1.272) | 0.522 |
| Skewness | 2.387 (1.768,3.966) | 1.871 (1.077,4.699) | 0.501 |
| Kurtosis | 9.323 (4.566,24.841) | 5.336 (1.108,55.734) | 0.459 |
| Uniformity | 0.251±0.220 | 0.328 (0.068,0.502) | 0.522 |
| Energy | 0.016 (0.014,0.038) | 0.018 (0.013,0.033) | 0.840 |
| Entropy | 6.048±0.797 | 6.125±0.817 | 0.783 |
| Quantity5 | 0.291±0.274 | 0.122 (0.034,0.369) | 0.459 |
| Quantity10 | 0.452±0.351 | 0.256 (0.096,0.520) | 0.281 |
| Quantity25 | 0.669±0.448 | 0.463 (0.317,0.787) | 0.334 |
| Quantity50 | 0.967±0.601 | 0.941 (0.577,1.248) | 0.522 |
| Quantity75 | 1.397±0.855 | 1.408 (0.740,1.525) | 0.590 |
| Quantity90 | 1.961±1.217 | 1.496 (1.039,2.382) | 0.686 |
| Quantity95 | 2.500±1.530 | 1.716 (1.269,3.145) | 0.544 |

**Supplementary table 1.8 Differences of ETM V_e_ perfusion histogram parameters between PD-L1 negative and positive expression groups**

| **Parameters** | **PD-L1 negative (n=14)** | **PD-L1 positive (n=21)** | ***P* value** |
| --- | --- | --- | --- |
| Meanvalue | 0.148 (0.114,0.443) | 0.172 (0.117,0.318) | 0.736 |
| Skewness | 2.445 (0.113,4.389) | 2.815±2.457 | 0.522 |
| Kurtosis | 34.931 (7.154,53.223) | 35.616 (3.849,74.731) | 0.973 |
| Uniformity | 0.447 (-0.125,0.604) | 0.217 (-0.538,0.499) | 0.266 |
| Energy | 0.014 (0.008,0.024) | 0.024 (0.008,0.030) | 0.381 |
| Entropy | 6.398±1.017 | 6.047±1.096 | 0.347 |
| Quantity5 | 0.043 (0.015,0.109) | 0.043 (0.014,0.069) | 0.736 |
| Quantity10 | 0.054 (0.028,0.172) | 0.064 (0.021,0.094) | 0.711 |
| Quantity25 | 0.080 (0.066,0.294) | 0.099 (0.037,0.171) | 0.637 |
| Quantity50 | 0.119 (0.090,0.410) | 0.132 (0.062,0.236) | 0.480 |
| Quantity75 | 0.164 (0.131,0.490) | 0.165 (0.092,0.361) | 0.439 |
| Quantity90 | 0.224 (0.160,0.578) | 0.191 (0.131,0.453) | 0.381 |
| Quantity95 | 0.262 (0.182,0.635) | 0.224 (0.154,0.502) | 0.346 |

**Supplementary table 1.9 Differences of ETM V_p_ perfusion histogram parameters between PD-L1 negative and positive expression groups**

| **Parameters** | **PD-L1 negative (n=14)** | **PD-L1 positive (n=21)** | ***P* value** |
| --- | --- | --- | --- |
| Meanvalue | 0.002 (0.001,0.008) | 0.001 (0.000,0.003) | 0.201 |
| Skewness | 5.715±3.884 | 4.896 (3.099,7.751) | 0.920 |
| Kurtosis | 35.923 (10.591,59.002) | 28.627 (10.259,76.682) | 0.866 |
| Uniformity | -2.300 (-3.562,-1.149) | -2.072 (-4.015,-1.147) | 0.973 |
| Energy | 0.619±0.290 | 0.632±0.270 | 0.890 |
| Entropy | 1.754 (0.906,2.964) | 2.812 (0.731,3.050) | 0.866 |
| Quantity5 | 0.000 (0.000,0.000) | 0.000 (0.000,0.000) | 0.099 |
| Quantity10 | 0.000 (0.000,0.000) | 0.000 (0.000,0.000) | 0.092 |
| Quantity25 | 0.000 (0.000,0.001) | 0.000 (0.000,0.000) | 0.138 |
| Quantity50 | 0.000 (0.000,0.002) | 0.000 (0.000,0.000) | 0.138 |
| Quantity75 | 0.001 (0.000,0.003) | 0.000 (0.000,0.002) | 0.312 |
| Quantity90 | 0.004 (0.001,0.015) | 0.003 (0.000,0.008) | 0.522 |
| Quantity95 | 0.010 (0.003,0.038) | 0.008 (0.002,0.013) | 0.252 |

**Supplementary table 2.1　Differences in ECM F_p_ perfusion histogram parameters between groups with weak and strong PD-L1 expression**

| **Parameters** | **Weak PD-L1 expression (n=25)** | **Strong PD-L1 expression (n=10)** | ***P* value** |
| --- | --- | --- | --- |
| Meanvalue | 0.159 (0.075,0.376) | 0.091 (0.056,0.118) | 0.108 |
| Skewness | 0.943 (0.572,1.449) | 0.615 (0.501,3.190) | 0.913 |
| Kurtosis | 1.269 (0.077,6.152) | 0.188 (-0.074,17.030) | 0.770 |
| Uniformity | 0.463±0.108 | 0.421 (0.175,0.526) | 0.144 |
| Energy | 0.010 (0.008,0.015) | 0.009 (0.007,0.039) | 0.942 |
| Entropy | 6.830±0.641 | 6.636±1.043 | 0.594 |
| Quantity5 | 0.044 (0.018,0.088) | 0.022 (0.017,0.037) | 0.273 |
| Quantity10 | 0.058 (0.028,0.120) | 0.032 (0.024,0.048) | 0.125 |
| Quantity25 | 0.098 (0.045,0.209) | 0.051 (0.037,0.072) | 0.068 |
| Quantity50 | 0.139 (0.073,0.354) | 0.087 (0.053,0.106) | 0.063 |
| Quantity75 | 0.185 (0.101,0.518) | 0.125 (0.071,0.154) | 0.086 |
| Quantity90 | 0.278 (0.125,0.653) | 0.162 (0.092,0.211) | 0.144 |
| Quantity95 | 0.377 (0.142,0.726) | 0.183 (0.111,0.250) | 0.144 |

**Supplementary table 2.2 Differences in ECM K_ep_ perfusion histogram parameters between groups with weak and strong PD-L1 expression**

| **Parameters** | **Weak PD-L1 expression (n=25)** | **Strong PD-L1 expression (n=10)** | ***P* value** |
| --- | --- | --- | --- |
| Meanvalue | 4.028±1.089 | 4.560±2.141 | 0.471 |
| Skewness | 0.004 (-0.434,0.364) | -0.238 (-1.494,0.364) | 0.381 |
| Kurtosis | -1.745 (-1.890,-1.481) | -0.744 (-1.766,3.979) | 0.053 |
| Uniformity | -0.066 (-0.097,0.282) | 0.232 (-0.230,0.519) | 0.361 |
| Energy | 0.287±0.170 | 0.281±0.201 | 0.926 |
| Entropy | 4.108±1.307 | 4.141±1.144 | 0.948 |
| Quantity5 | 0.003 (0.002,0.004) | 0.003 (0.002,0.005) | 0.770 |
| Quantity10 | 0.006 (0.005,0.009) | 0.006 (0.005,0.011) | 0.770 |
| Quantity25 | 0.014 (0.012,0.022) | 0.016 (0.012,0.027) | 0.798 |
| Quantity50 | 0.028 (0.024,0.504) | 0.048 (0.024,0.667) | 0.770 |
| Quantity75 | 0.968±0.711 | 0.884 (0.214,1.716) | 0.971 |
| Quantity90 | 2.375±1.393 | 2.619±1.857 | 0.674 |
| Quantity95 | 3.641±1.749 | 4.077±1.908 | 0.521 |

**Supplementary table 2.3 Differences in ECM K^trans^ perfusion histogram parameters between groups with weak and strong PD-L1 expression**

| **Parameters** | **Weak PD-L1 expression (n=25)** | **Strong PD-L1 expression (n=10)** | ***P* value** |
| --- | --- | --- | --- |
| Meanvalue | 0.226 (0.091,0.422) | 0.113 (0.062,0.128) | 0.080 |
| Skewness | 6.061±4.341 | 7.383 (3.533,17.406) | 0.307 |
| Kurtosis | 84.649 (4.449,118.004) | 156.659 (31.437,457.443) | 0.189 |
| Uniformity | 0.150 (-0.197,0.468) | 0.314 (-0.443,0.439) | 0.884 |
| Energy | 0.045 (0.021,0.073) | 0.070 (0.028,0.160) | 0.177 |
| Entropy | 4.790 (4.245,6.109) | 4.390±1.442 | 0.116 |
| Quantity5 | 0.049 (0.021,0.098) | 0.026 (0.020,0.039) | 0.243 |
| Quantity10 | 0.064 (0.031,0.127) | 0.039 (0.026,0.052) | 0.108 |
| Quantity25 | 0.105 (0.052,0.229) | 0.059 (0.040,0.077) | 0.063 |
| Quantity50 | 0.148 (0.083,0.388) | 0.100 (0.056,0.115) | 0.080 |
| Quantity75 | 0.219 (0.111,0.550) | 0.143 (0.075,0.161) | 0.074 |
| Quantity90 | 0.368 (0.145,0.684) | 0.184 (0.099,0.227) | 0.108 |
| Quantity95 | 0.428 (0.172,0.845) | 0.218 (0.122,0.295) | 0.108 |

**Supplementary table 2.4 Differences in ECM V_e_ perfusion histogram parameters between groups with weak and strong PD-L1 expression**

| **Parameters** | **Weak PD-L1 expression (n=25)** | **Strong PD-L1 expression (n=10)** | ***P* value** |
| --- | --- | --- | --- |
| Meanvalue | 0.397±0.143 | 0.335±0.241 | 0.459 |
| Skewness | 0.588 (0.045,0.981) | 0.960 (0.097,2.434) | 0.422 |
| Kurtosis | -1.488 (-1.737,-0.802) | 0.292(-1.904,4.953) | 0.535 |
| Uniformity | -0.167 (-0.431,0.128) | -0.517 (-1.514,0.096) | 0.273 |
| Energy | 0.128 (0.043,0.295) | 0.190±0.173 | 0.189 |
| Entropy | 4.508±1.351 | 3.372±1.959 | 0.057 |
| Quantity5 | 0.001 (0.000,0.002) | 0.001 (0.000,0.002) | 0.258 |
| Quantity10 | 0.001 (0.001,0.004) | 0.001 (0.001,0.005) | 0.273 |
| Quantity25 | 0.004 (0.002,0.010) | 0.003 (0.001,0.014) | 0.307 |
| Quantity50 | 0.009 (0.004,0.033) | 0.005 (0.003,0.048) | 0.201 |
| Quantity75 | 0.104 (0.044,0.151) | 0.008 (0.004,0.113) | 0.024* |
| Quantity90 | 0.317±0.188 | 0.091 (0.038,0.361) | 0.041* |
| Quantity95 | 0.462±0.203 | 0.327±0.289 | 0.125 |

* means P < 0.05

**Supplementary table 2.5 Differences in ECM V_p_ perfusion histogram parameters between groups with weak and strong PD-L1 expression**

| **Parameters** | **Weak PD-L1 expression (n=25)** | **Strong PD-L1 expression (n=10)** | ***P* value** |
| --- | --- | --- | --- |
| Meanvalue | 0.118 (0.087,0.272) | 0.104 (0.052,0.355) | 0.535 |
| Skewness | 0.675 (0.178,3.289) | 1.191 (0.199,4.751) | 0.635 |
| Kurtosis | 2.493 (-0.115,22.397) | 0.894 (-0.233,24.278) | 0.742 |
| Uniformity | 0.391 (0.047,0.544) | 0.243 (-0.296,0.455) | 0.258 |
| Energy | 0.011 (0.008,0.027) | 0.014 (0.007,0.033) | 0.715 |
| Entropy | 6.523±0.976 | 6.373±1.263 | 0.708 |
| Quantity5 | 0.012 (0.003,0.034) | 0.005 (0.002,0.028) | 0.361 |
| Quantity10 | 0.023 (0.008,0.071) | 0.013 (0.004,0.042) | 0.243 |
| Quantity25 | 0.065 (0.032,0.157) | 0.039 (0.011,0.128) | 0.258 |
| Quantity50 | 0.112 (0.072,0.276) | 0.065 (0.025,0.220) | 0.154 |
| Quantity75 | 0.157 (0.110,0.373) | 0.101 (0.042,0.313) | 0.189 |
| Quantity90 | 0.198 (0.135,0.454) | 0.140 (0.074,0.432) | 0.273 |
| Quantity95 | 0.238 (0.147,0.495) | 0.179 (0.089,0.531) | 0.342 |

**Supplementary table 2.6 Differences in ETM K^trans^ perfusion histogram parameters between groups with weak and strong PD-L1 expression**

| **Parameters** | **Weak PD-L1 expression (n=25)** | **Strong PD-L1 expression (n=10)** | ***P* value** |
| --- | --- | --- | --- |
| Meanvalue | 0.149 (0.065,0.384) | 0.060 (0.049,0.116) | 0.074 |
| Skewness | 0.988 (0.484,2.202) | 1.965±1.186 | 0.243 |
| Kurtosis | 0.855 (0.019,12.243) | 7.136 (0.079,17.790) | 0.307 |
| Uniformity | 0.438 (0.361,0.501) | 0.340 (0.055,0.501) | 0.214 |
| Energy | 0.012 (0.008,0.021) | 0.024±0.017 | 0.154 |
| Entropy | 6.533±0.806 | 6.115±0.881 | 0.186 |
| Quantity5 | 0.034 (0.011,0.081) | 0.020 (0.005,0.026) | 0.116 |
| Quantity10 | 0.048 (0.021,0.127) | 0.025 (0.009,0.037) | 0.080 |
| Quantity25 | 0.081 (0.038,0.237) | 0.036 (0.020,0.057) | 0.031* |
| Quantity50 | 0.125 (0.061,0.362) | 0.055 (0.039,0.101) | 0.045* |
| Quantity75 | 0.195 (0.088,0.507) | 0.078 (0.058,0.153) | 0.068 |
| Quantity90 | 0.270 (0.107,0.669) | 0.106 (0.091,0.189) | 0.134 |
| Quantity95 | 0.341 (0.121,0.760) | 0.146 (0.108,0.246) | 0.177 |

* means P < 0.05

**Supplementary table 2.7 Differences in ETM K_ep_ perfusion histogram parameters between groups with weak and strong PD-L1 expression**

| **Parameters** | **Weak PD-L1 expression (n=25)** | **Strong PD-L1 expression (n=10)** | ***P* value** |
| --- | --- | --- | --- |
| Meanvalue | 0.982 (0.686,1.284) | 1.203±0.891 | 0.827 |
| Skewness | 2.608 (1.378,4.252) | 1.777 (0.825,5.161) | 0.422 |
| Kurtosis | 9.785 (3.477,27.109) | 5.275 (0.809,63.830) | 0.342 |
| Uniformity | 0.355 (0.225,0.465) | 0.158 (-0.381,0.433) | 0.165 |
| Energy | 6.142 (5.432,6.743) | 6.460 (5.489,6.600) | 0.584 |
| Entropy | 6.105±0.758 | 6.067±0.933 | 0.901 |
| Quantity5 | 0.222 (0.100,0.397) | 0.046 (0.014,0.293) | 0.074 |
| Quantity10 | 0.402 (0.212,0.570) | 0.105 (0.032,0.395) | 0.049* |
| Quantity25 | 0.636±0.355 | 0.324 (0.132,0.680) | 0.100 |
| Quantity50 | 0.910±0.479 | 0.594 (0.468,1.132) | 0.361 |
| Quantity75 | 1.232 (0.796,1.563) | 1.490±1.116 | 0.827 |
| Quantity90 | 1.496 (1.072,2.100) | 2.375±1.621 | 0.422 |
| Quantity95 | 1.872 (1.399,2.636) | 2.882±1.941 | 0.443 |

* means P < 0.05

**Supplementary table 2.8 Differences in ETM V_e_ perfusion histogram parameters between groups with weak and strong PD-L1 expression**

| **Parameters** | **Weak PD-L1 expression (n=25)** | **Strong PD-L1 expression (n=10)** | ***P* value** |
| --- | --- | --- | --- |
| Meanvalue | 0.150 (0.114,0.318) | 0.188 (0.122,0.371) | 0.609 |
| Skewness | 2.839±2.631 | 1.644 (0.484,4.095) | 0.535 |
| Kurtosis | 10.136 (1.265,43.360) | 1.105 (-0.135,16.916) | 0.125 |
| Uniformity | 0.440 (-0.141,0.586) | 0.159 (-0.576,0.429) | 0.273 |
| Energy | 0.016 (0.008,0.030) | 0.025 (0.010,0.036) | 0.381 |
| Entropy | 6.271±1.053 | 5.977±1.118 | 0.469 |
| Quantity5 | 0.044 (0.018,0.073) | 0.043±0.041 | 0.324 |
| Quantity10 | 0.064 (0.032,0.106) | 0.036 (0.011,0.099) | 0.258 |
| Quantity25 | 0.099 (0.064,0.195) | 0.055 (0.024,0.147) | 0.134 |
| Quantity50 | 0.132 (0.090,0.314) | 0.086 (0.034,0.233) | 0.108 |
| Quantity75 | 0.172 (0.123,0.411) | 0.120 (0.051,0.321) | 0.154 |
| Quantity90 | 0.220 (0.150,0.492) | 0.167 (0.119,0.441) | 0.273 |
| Quantity95 | 0.254 (0.175,0.545) | 0.209 (0.151,0.540) | 0.401 |

**Supplementary table 2.9 Differences in ETM V_p_ perfusion histogram parameters between groups with weak and strong PD-L1 expression**

| **Parameters** | **Weak PD-L1 expression (n=25)** | **Strong PD-L1 expression (n=10)** | ***P* value** |
| --- | --- | --- | --- |
| Meanvalue | 0.002 (0.001,0.003) | 0.001 (0.000,0.005) | 0.201 |
| Skewness | 4.925 (3.099,6.286) | 7.164±6.553 | 0.920 |
| Kurtosis | 30.018 (10.807,54.708) | 49.435 (2.019,149.352) | 0.866 |
| Uniformity | -2.072 (-3.320,-1.217) | -3.265 (-5.235,-0.496) | 0.973 |
| Energy | 0.670 (0.511,0.834) | 0.607±0.349 | 0.866 |
| Entropy | 1.812 (0.955,2.698) | 2.440±2.360 | 0.866 |
| Quantity5 | 0.000 (0.000,0.000) | 0.000 (0.000,0.000) | 0.099 |
| Quantity10 | 0.000 (0.000,0.000) | 0.000 (0.000,0.000) | 0.092 |
| Quantity25 | 0.000 (0.000,0.000) | 0.000 (0.000,0.001) | 0.138 |
| Quantity50 | 0.000 (0.000,0.010) | 0.000 (0.000,0.003) | 0.138 |
| Quantity75 | 0.001 (0.000,0.002) | 0.000 (0.000,0.007) | 0.312 |
| Quantity90 | 0.004 (0.001,0.009) | 0.001 (0.000,0.012) | 0.522 |
| Quantity95 | 0.008 (0.003,0.017) | 0.006 (0.001,0.016) | 0.252 |

**Supplementary table 3.1 Differences in perfusion histogram parameters of ECM F_p_ between PD-L1 non-expression, low expression and high expression groups**

| **Parameters** | **PD-L1 non-expression (n=14)** | **PD-L1 low expression (n=11)** | **PD-L1 high expression (n=10)** | ***P* value** |
| --- | --- | --- | --- | --- |
| Meanvalue | 0.145 (0.076,0.595) | 0.200±0.139 | 0.091 (0.056,0.118) | 0.271 |
| Skewness | 1.013 (0.358,1.831) | 1.207±0.852 | 0.615 (0.501,3.190) | 0.992 |
| Kurtosis | 2.295 (-0.455,8.050) | 0.872 (0.631,4.092) | 0.188 (-0.074,17.030) | 0.954 |
| Uniformity | 0.461±0.098 | 0.465±0.124 | 0.421 (0.175,0.526) | 0.330 |
| Energy | 0.011 (0.006,0.017) | 0.010±0.003 | 0.009 (0.007,0.039) | 0.994 |
| Entropy | 6.773±0.785 | 6.904±0.420 | 6.636±1.043 | 0.739 |
| Quantity5 | 0.030 (0.017,0.172) | 0.049±0.031 | 0.022 (0.017,0.037) | 0.544 |
| Quantity10 | 0.052 (0.027,0.241) | 0.072±0.044 | 0.032 (0.024,0.048) | 0.308 |
| Quantity25 | 0.086 (0.045,0.388) | 0.123±0.083 | 0.051 (0.037,0.072) | 0.188 |
| Quantity50 | 0.127 (0.074,0.541) | 0.186±0.132 | 0.087 (0.053,0.106) | 0.174 |
| Quantity75 | 0.181 (0.103,0.730) | 0.262±0.189 | 0.125 (0.071,0.154) | 0.222 |
| Quantity90 | 0.259 (0.128,0.994) | 0.341±0.242 | 0.162 (0.092,0.211) | 0.343 |
| Quantity95 | 0.321 (0.146,1.159) | 0.393±0.273 | 0.183 (0.111,0.251) | 0.342 |

**Supplementary table 3.2 Differences in perfusion histogram parameters of ECM K_ep_ between PD-L1 non-expression, low expression and high expression groups**

| **Parameters** | **PD-L1 non-expression (n=14)** | **PD-L1 low expression (n=11)** | **PD-L1 high expression (n=10)** | ***P* value** |
| --- | --- | --- | --- | --- |
| Meanvalue | 4.405±1.124 | 3.476±1.031 | 4.560±2.141 | 0.087 |
| Skewness | 0.904 (0.358,1.443) | 0.895 (0.667,1.386) | 0.613 (0.438,2.449) | 0.069 |
| Kurtosis | 1.367 (-0.395,5.858) | 0.872 (0.631,4.092) | 0.258 (-0.008,7.471) | 0.152 |
| Uniformity | 0.462±0.098 | 0.465±0.124 | 0.456 (0.189,0.538) | 0.095 |
| Energy | 0.255±0.163 | 0.327±0.177 | 0.281±0.201 | 0.608 |
| Entropy | 4.363±1.279 | 3.784±1.330 | 4.141±1.439 | 0.569 |
| Quantity5 | 0.033 (0.017,0.108) | 0.049±0.031 | 0.022 (0.020,0.037) | 0.626 |
| Quantity10 | 0.048 (0.027,0.168) | 0.072±0.044 | 0.034 (0.026,0.049) | 0.626 |
| Quantity25 | 0.086 (0.045,0.298) | 0.123±0.083 | 0.053 (0.037,0.073) | 0.599 |
| Quantity50 | 0.134 (0.074,0.425) | 0.186±0.132 | 0.099 (0.055,0.108) | 0.603 |
| Quantity75 | 1.150±0.781 | 0.736±0.562 | 0.135 (0.073,0.174) | 0.463 |
| Quantity90 | 2.747±1.508 | 1.902±1.122 | 2.619±1.857 | 0.360 |
| Quantity95 | 4.111±1.787 | 3.043±1.578 | 4.077±1.908 | 0.273 |

**Supplementary table 3.3 Differences in perfusion histogram parameters of ECM K^trans^ between PD-L1 non-expression, low expression and high expression groups**

| **Parameters** | **PD-L1 non-expression (n=14)** | **PD-L1 low expression (n=11)** | **PD-L1 high expression (n=10)** | ***P* value** |
| --- | --- | --- | --- | --- |
| Meanvalue | 0.178 (0.094,0.624) | 0.246±0.157 | 0.113 (0.062,0.128) | 0.214 |
| Skewness | 5.388±4.820 | 6.917±3.687 | 7.383 (3.533,17.406) | 0.403 |
| Kurtosis | 63.217 (0.245,123.217) | 93.066±71.027 | 156.659 (31.437,457.443) | 0.301 |
| Uniformity | 0.329 (-0.272,0.513) | 0.111 (-0.063,0.244) | 0.314 (-0.443,0.439) | 0.775 |
| Energy | 0.053±0.044 | 0.048±0.026 | 0.070 (0.028,0.160) | 0.401 |
| Entropy | 5.330±1.390 | 4.790 (4.229,5.359) | 4.390±1.442 | 0.261 |
| Quantity5 | 0.032 (0.021,0.204) | 0.058±0.034 | 0.026 (0.020,0.039) | 0.475 |
| Quantity10 | 0.056 (0.031,0.276) | 0.084±0.052 | 0.039 (0.026,0.052) | 0.275 |
| Quantity25 | 0.092 (0.052,0.420) | 0.138±0.094 | 0.059 (0.040,0.077) | 0.177 |
| Quantity50 | 0.135 (0.085,0.568) | 0.209±0.145 | 0.100 (0.056,0.115) | 0.214 |
| Quantity75 | 0.205 (0.113,0.755) | 0.300±0.207 | 0.143 (0.075,0.161) | 0.199 |
| Quantity90 | 0.284 (0.148,1.021) | 0.406±0.260 | 0.184 (0.099,0.227) | 0.269 |
| Quantity95 | 0.410 (0.179,1.192) | 0.498±0.323 | 0.218 (0.122,0.295) | 0.269 |

**Supplementary table 3.4 Differences in perfusion histogram parameters of ECM V_e_ between PD-L1 non-expression, low expression and high expression groups**

| **Parameters** | **PD-L1 non-expression (n=14)** | **PD-L1 low expression (n=11)** | **PD-L1 high expression (n=10)** | ***P* value** |
| --- | --- | --- | --- | --- |
| Meanvalue | 0.349±0.127 | 0.459±0.142 | 0.335±0.241 | 0.158 |
| Skewness | 0.836±0.670 | 0.369 (-0.507,0.459) | 0.960 (0.097,2.434) | 0.148 |
| Kurtosis | -1.293 (-1.641,0.048) | -1.690 (-1.748,-1.348) | 0.292 (-1.904,4.953) | 0.479 |
| Uniformity | -0.327 (-0.619,0.006) | -0.083 (-0.170,0.268) | -0.517 (-1.514,0.096) | 0.152 |
| Energy | 0.129 (0.033,0.281) | 0.179±0.138 | 0.355±0.274 | 0.419 |
| Entropy | 4.443±1.456 | 4.591±1.269 | 3.372±1.959 | 0.164 |
| Quantity5 | 0.001 (0.000,0.008) | 0.001±0.0007 | 0.001 (0.000,0.002) | 0.522 |
| Quantity10 | 0.001 (0.001,0.016) | 0.002±0.001 | 0.001 (0.001,0.005) | 0.541 |
| Quantity25 | 0.004 (0.002,0.030) | 0.004 (0.002,0.008) | 0.003 (0.001,0.014) | 0.592 |
| Quantity50 | 0.009 (0.005,0.048) | 0.009 (0.004,0.031) | 0.005 (0.003,0.048) | 0.437 |
| Quantity75 | 0.090 (0.023,0.137) | 0.104 (0.049,0.254) | 0.008 (0.004,0.113) | 0.044* |
| Quantity90 | 0.272±0.170 | 0.374±0.203 | 0.091 (0.038,0.361) | 0.072 |
| Quantity95 | 0.413±0.198 | 0.526±0.200 | 0.327±0.289 | 0.149 |

* means P < 0.05

**Supplementary table 3.5 Differences in perfusion histogram parameters of ECM V_p_ between PD-L1 non-expression, low expression and high expression groups**

| **Parameters** | **PD-L1 non-expression (n=14)** | **PD-L1 low expression (n=11)** | **PD-L1 high expression (n=10)** | ***P* value** |
| --- | --- | --- | --- | --- |
| Meanvalue | 0.114 (0.088,0.373) | 0.124 (0.071,0.263) | 0.104 (0.052,0.355) | 0.769 |
| Skewness | 1.217 (0.138,4.433) | 0.626 (0.146,1.968) | 1.191 (0.199,4.751) | 0.746 |
| Kurtosis | 4.305 (0.015,19.960) | 0.988 (-0.353,5.243) | 0.894 (-0.233,24.278) | 0.696 |
| Uniformity | 0.392 (-0.193,0.577) | 0.387 (0.094,0.523) | 0.243 (-0.296,0.455) | 0.524 |
| Energy | 0.012 (0.007,0.031) | 0.011 (0.008,0.023) | 0.014 (0.007,0.033) | 0.927 |
| Entropy | 6.432±1.095 | 6.639±0.836 | 6.373±1.263 | 0.833 |
| Quantity5 | 0.018 (0.002,0.061) | 0.006 (0.003,0.033) | 0.005 (0.002,0.028) | 0.499 |
| Quantity10 | 0.033 (0.007,0.130) | 0.018 (0.008,0.057) | 0.013 (0.004,0.042) | 0.370 |
| Quantity25 | 0.063 (0.035,0.262) | 0.065 (0.023,0.142) | 0.039 (0.011,0.128) | 0.483 |
| Quantity50 | 0.102 (0.078,0.369) | 0.127 (0.060,0.265) | 0.065 (0.025,0.220) | 0.363 |
| Quantity75 | 0.146 (0.113,0.451) | 0.238±0.190 | 0.101 (0.042,0.313) | 0.407 |
| Quantity90 | 0.199 (0.148,0.531) | 0.294±0.225 | 0.140 (0.074,0.432) | 0.489 |
| Quantity95 | 0.243 (0.168,0.591) | 0.326±0.248 | 0.179 (0.089,0.531) | 0.517 |

**Supplementary table 3.6 Differences in perfusion histogram parameters of ETM K^trans^ between PD-L1 non-expression, low expression and high expression groups**

| **Parameters** | **PD-L1 non-expression (n=14)** | **PD-L1 low expression (n=11)** | **PD-L1 high expression (n=10)** | ***P* value** |
| --- | --- | --- | --- | --- |
| Meanvalue | 0.145 (0.065,0.752) | 0.179±0.132 | 0.060 (0.049,0.116) | 0.182 |
| Skewness | 1.167 (0.539,3.117) | 0.751 (0.454,1.827) | 1.965±1.186 | 0.325 |
| Kurtosis | 3.347 (-0.204,21.098) | 0.723 (0.165,11.990) | 7.136 (0.079,17.790) | 0.502 |
| Uniformity | 0.411 (0.343,0.520) | 0.443±0.139 | 0.340 (0.055,0.501) | 0.440 |
| Energy | 0.019±0.012 | 0.008 (0.007,0.017) | 0.024±0.017 | 0.113 |
| Entropy | 6.332±0.888 | 7.134 (6.122,7.292) | 6.115±0.881 | 0.138 |
| Quantity5 | 0.029 (0.014,0.150) | 0.036±0.027 | 0.020 (0.005,0.026) | 0.256 |
| Quantity10 | 0.044 (0.020,0.255) | 0.057±0.041 | 0.025 (0.009,0.037) | 0.205 |
| Quantity25 | 0.079 (0.038,0.473) | 0.106±0.078 | 0.036 (0.020,0.057) | 0.091 |
| Quantity50 | 0.124 (0.061,0.665) | 0.167±0.124 | 0.055 (0.039,0.101) | 0.125 |
| Quantity75 | 0.194 (0.089,0.901) | 0.239±0.182 | 0.078 (0.058,0.153) | 0.175 |
| Quantity90 | 0.266 (0.108,1.245) | 0.312±0.238 | 0.320 (0.122,0.209) | 0.306 |
| Quantity95 | 0.320 (0.122,1.532) | 0.360±0.269 | 0.146 (0.108,0.246) | 0.376 |

**Supplementary table 3.7 Differences in perfusion histogram parameters of ETM K_ep_ between PD-L1 non-expression, low expression and high expression groups**

| **Parameters** | **PD-L1 non-expression (n=14)** | **PD-L1 low expression (n=11)** | **PD-L1 high expression (n=10)** | ***P* value** |
| --- | --- | --- | --- | --- |
| Meanvalue | 1.152±0.714 | 0.913±0.272 | 1.203±0.891 | 0.567 |
| Skewness | 1.931 (1.042,3.374) | 3.415±2.826 | 1.684 (0.988,2.908) | 0.692 |
| Kurtosis | 6.857 (1.811,21.372) | 18.228 (2.690,82.190) | 5.213 (0.868,10.449) | 0.611 |
| Uniformity | 0.251±0.220 | 0.389±0.180 | 0.094±0.407 | 0.109 |
| Energy | 0.015 (0.011,0.028) | 0.019±0.009 | 0.018 (0.014,0.066) | 0.733 |
| Entropy | 6.048±0.797 | 6.178±0.737 | 6.067±0.933 | 0.918 |
| Quantity5 | 0.291±0.274 | 0.252±0.173 | 0.038 (0.007,0.228) | 0.197 |
| Quantity10 | 0.452±0.351 | 0.383±0.179 | 0.048 (0.014,0.296) | 0.143 |
| Quantity25 | 0.669±0.448 | 0.594±0.199 | 0.287 (0.090,0.570) | 0.256 |
| Quantity50 | 0.967±0.601 | 0.838±0.264 | 0.562 (0.431,0.999) | 0.647 |
| Quantity75 | 1.397±0.855 | 1.116±0.326 | 1.490±1.116 | 0.553 |
| Quantity90 | 1.961±1.217 | 1.456±0.397 | 2.375±1.621 | 0.458 |
| Quantity95 | 2.500±1.530 | 1.752±0.510 | 2.882±1.941 | 0.376 |

**Supplementary table 3.8 Differences in perfusion histogram parameters of ETM V_e_ between PD-L1 non-expression, low expression and high expression groups**

| **Parameters** | **PD-L1 non-expression (n=14)** | **PD-L1 low expression (n=11)** | **PD-L1 high expression (n=10)** | ***P* value** |
| --- | --- | --- | --- | --- |
| Meanvalue | 0.148 (0.114,0.443) | 0.161 (0.109,0.303) | 0.188 (0.122,0.371) | 0.874 |
| Skewness | 2.445 (0.113,4.389) | 3.454±2.473 | 1.644 (0.484,4.095) | 0.440 |
| Kurtosis | 7.154 (0.262,34.931) | 18.618 (2.348,51.460) | 1.105 (-0.135,16.916) | 0.208 |
| Uniformity | 0.447 (-0.125,0.604) | 0.412 (-0.535,0.526) | 0.159 (-0.576,0.429) | 0.448 |
| Energy | 0.014 (0.008,0.024) | 0.023±0.017 | 0.025 (0.010,0.036) | 0.603 |
| Entropy | 6.398±1.017 | 6.110±1.125 | 5.977±1.118 | 0.622 |
| Quantity5 | 0.043 (0.015,0.109) | 0.056 (0.019,0.072) | 0.043±0.041 | 0.603 |
| Quantity10 | 0.054 (0.028,0.172) | 0.071 (0.034,0.099) | 0.036 (0.011,0.099) | 0.511 |
| Quantity25 | 0.080 (0.066,0.294) | 0.115 (0.053,0.178) | 0.055 (0.024,0.147) | 0.306 |
| Quantity50 | 0.119 (0.090,0.410) | 0.158 (0.073,0.311) | 0.086 (0.034,0.233) | 0.272 |
| Quantity75 | 0.164 (0.131,0.490) | 0.271±0.204 | 0.120 (0.051,0.321) | 0.362 |
| Quantity90 | 0.224 (0.160,0.578) | 0.326±0.239 | 0.167 (0.119,0.441) | 0.514 |
| Quantity95 | 0.262 (0.182,0.635) | 0.363±0.259 | 0.209 (0.151,0.540) | 0.589 |

**Supplementary table 3.9 Differences in perfusion histogram parameters of ETM V_p_ between PD-L1 non-expression, low expression and high expression groups**

| **Parameters** | **PD-L1 non-expression (n=14)** | **PD-L1 low expression (n=11)** | **PD-L1 high expression (n=10)** | ***P* value** |
| --- | --- | --- | --- | --- |
| Meanvalue | 0.002 (0.001,0.009) | 0.002±0.001 | 0.001 (0.000,0.005) | 0.379 |
| Skewness | 5.715±3.884 | 3.634 (3.150,5.498) | 7.164±6.553 | 0.755 |
| Kurtosis | 35.923 (10.591,59.002) | 14.795 (10.839,45.010) | 49.435 (2.019,149.352) | 0.773 |
| Uniformity | -2.300 (-3.562,-1.149) | -1.939 (-2.617,-1.260) | -3.265 (-5.235,-0.496) | 0.846 |
| Energy | 0.619±0.190 | 0.656±0.186 | 0.607±0.349 | 0.915 |
| Entropy | 1.754 (0.906,2.964) | 1.904±1.036 | 2.440±2.360 | 0.967 |
| Quantity5 | 0.000 (0.000,0.000) | 0.000 (0.000,0.000) | 0.000 (0.000,0.000) | 0.247 |
| Quantity10 | 0.000 (0.000,0.000) | 0.000 (0.000,0.000) | 0.000 (0.000,0.000) | 0.235 |
| Quantity25 | 0.000 (0.000,0.001) | 0.000 (0.000,0.000) | 0.000 (0.000,0.001) | 0.305 |
| Quantity50 | 0.000 (0.000,0.002) | 0.000 (0.000,0.000) | 0.000 (0.000,0.003) | 0.305 |
| Quantity75 | 0.001 (0.000,0.003) | 0.000 (0.000,0.001) | 0.000 (0.000,0.007) | 0.511 |
| Quantity90 | 0.004 (0.001,0.015) | 0.003 (0.002,0.006) | 0.001 (0.000,0.012) | 0.665 |
| Quantity95 | 0.010 (0.003,0.038) | 0.009±0.008 | 0.006 (0.001,0.016) | 0.457 |
